# Human papillomavirus vaccination for adults aged 30 to 45 years in the United States: A cost-effectiveness analysis

**DOI:** 10.1371/journal.pmed.1003534

**Published:** 2021-03-11

**Authors:** Jane J. Kim, Kate T. Simms, James Killen, Megan A. Smith, Emily A. Burger, Stephen Sy, Catherine Regan, Karen Canfell

**Affiliations:** 1 Department of Health Policy and Management, Center for Health Decision Science, Harvard T.H. Chan School of Public Health, Boston, Massachusetts, United States of America; 2 Cancer Research Division, Cancer Council New South Wales, Sydney, Australia; 3 School of Public Health, University of Sydney, Sydney, Australia; 4 Department of Health Management and Health Economics, University of Oslo, Oslo, Norway; UTHealth, UNITED STATES

## Abstract

**Background:**

A nonavalent human papillomavirus (HPV) vaccine has been licensed for use in women and men up to age 45 years in the United States. The cost-effectiveness of HPV vaccination for women and men aged 30 to 45 years in the context of cervical cancer screening practice was evaluated to inform national guidelines.

**Methods and findings:**

We utilized 2 independent HPV microsimulation models to evaluate the cost-effectiveness of extending the upper age limit of HPV vaccination in women (from age 26 years) and men (from age 21 years) up to age 30, 35, 40, or 45 years. The models were empirically calibrated to reflect the burden of HPV and related cancers in the US population and used standardized inputs regarding historical and future vaccination uptake, vaccine efficacy, cervical cancer screening, and costs. Disease outcomes included cervical, anal, oropharyngeal, vulvar, vaginal, and penile cancers, as well as genital warts. Both models projected higher costs and greater health benefits as the upper age limit of HPV vaccination increased. Strategies of vaccinating females and males up to ages 30, 35, and 40 years were found to be less cost-effective than vaccinating up to age 45 years, which had an incremental cost-effectiveness ratio (ICER) greater than a commonly accepted upper threshold of $200,000 per quality-adjusted life year (QALY) gained. When including all HPV-related outcomes, the ICER for vaccinating up to age 45 years ranged from $315,700 to $440,600 per QALY gained. Assumptions regarding cervical screening compliance, vaccine costs, and the natural history of noncervical HPV-related cancers had major impacts on the cost-effectiveness of the vaccination strategies. Key limitations of the study were related to uncertainties in the data used to inform the models, including the timing of vaccine impact on noncervical cancers and vaccine efficacy at older ages.

**Conclusions:**

Our results from 2 independent models suggest that HPV vaccination for adult women and men aged 30 to 45 years is unlikely to represent good value for money in the US.

## Abstract 2

Jane Kim and co-workers estimate the potential cost-effectiveness of papillomavirus vaccination for adults aged 30-45 years in the United States.

## Author summary

**Why was this study done?:**

- Policies for increasing the upper age limit for human papillomavirus (HPV) vaccination to women and men up to age 45 years are being considered in several countries, but the public health value of such policies is uncertain.
- An important background consideration is to understand the impact and costs of HPV vaccination in the context of existing cervical cancer screening.
- This study was conducted to directly inform the deliberations of the Advisory Committee on Immunization Practices (ACIP) to guide HPV vaccination policy for women and men in the United States.

**What did the researchers do and find?:**

- Two modeling groups that are part of the Cancer Intervention and Surveillance Modeling Network (CISNET) used independent mathematical models calibrated to the US population to evaluate the cost-effectiveness of extending HPV vaccination in females and males up to ages 30, 35, 40, or 45 years.
- We conducted the analysis in the context of HPV transmission dynamics, historical HPV vaccination uptake, and detailed cervical cancer screening practice patterns in the US population.
- We evaluated cost and health outcomes of HPV vaccination up to age 45 years for cervical disease, as well as 6 other disease outcomes (5 noncervical HPV-related cancers and genital warts) in both women and men.
- Findings from both models suggest that at current prices, HPV vaccination beyond age 26 years is not cost-effective, even under a range of sensitivity analyses and favorable assumptions regarding HPV vaccination effectiveness and costs.

**What do these findings mean?:**

- HPV vaccination of women and men aged 30 to 45 years provides limited health benefit at the population level, at a substantial cost (at current HPV vaccine prices).
- Public health decision-makers considering the option to extend HPV vaccination to adults up to age 45 years should consider this evaluation of the value—and the opportunity costs—of adopting such a policy.

## Introduction

In the United States, routine vaccination against human papillomavirus (HPV) has been recommended for girls and young women up to age 26 years since 2006 and boys and young men up to age 21 years since 2009 [1,2]. Since then, with continually emerging data from vaccine trials, HPV vaccination policy has been revised to include fewer (two) doses for individuals up to age 14 years, as well as include a new HPV vaccine. Initially, 2 HPV vaccines were available, targeting HPV-16 and HPV-18, the 2 most oncogenic types that are responsible for roughly 70% of cervical cancers, as well as proportions of anal, vulvar, vaginal, penile, and oropharyngeal cancers: a bivalent vaccine (HPV-2) targeting HPV-16/18 only, and a quadrivalent vaccine (HPV-4) additionally targeting HPV-6/11, responsible for most genital warts and recurrent respiratory papillomatosis (RRP). More recently, a broad-spectrum nonavalent vaccine (HPV-9) was licensed that targets 7 high-risk HPV genotypes (HPV-16/18/31/33/45/52/58) that account for up to 90% of cervical cancer cases [3], as well as HPV-6/11. All current HPV vaccines appear highly efficacious in preventing vaccine-targeted HPV infections and precancerous lesions in individuals not previously infected with these types [4–12].

Since the vaccines are most efficacious prior to HPV exposure, guidelines have prioritized targeting young adolescents 11 to 12 years of age, aiming to reach individuals prior to sexual initiation [1,13,14]. However, evidence supporting vaccine efficacy in previously unexposed older populations and the recent US licensure of HPV-9 up to age 45 years have revived the question of the optimal age limit for HPV vaccination. In June 2019, the Advisory Committee on Immunization Practices (ACIP), which advises the US Centers for Disease Control and Prevention (CDC), voted to harmonize the upper age limit for catch-up HPV vaccination across genders to age 26 years but did not expand catch-up recommendations beyond age 26 years; instead, ACIP stated that HPV vaccination between ages 27 to 45 years be based on shared clinical decision-making [15].

Estimating the health impact of HPV vaccination in a population is complex given the decades-long natural history process of HPV infection to cancer. Long-run evaluations of the impact and value (i.e., cost-effectiveness) of different HPV vaccination strategies is further complicated when considering the extensive cervical cancer screening program in the US. Mathematical models that synthesize the best-available data while ensuring consistency with epidemiological observations can project outcomes beyond those reported in short-term clinical studies and provide insight into key drivers of impact and cost-effectiveness. In this analysis, leveraging a comparative modeling collaboration, we evaluated the cost-effectiveness of extending the upper age limit of HPV vaccination in women and men up to age 45 years to inform the deliberations of the ACIP. We explicitly considered HPV transmission dynamics, historical uptake and recommended doses of different HPV vaccines over time, detailed algorithms of cervical cancer screening, and the potential benefits of preventing noncervical HPV-related outcomes in the US population.

## Methods

### Analytic overview

This analysis, which consisted of a model-based evaluation using well-validated, existing modeling platforms, was conducted to help inform policy decisions that were deliberated at ACIP meetings held in February and June 2019 [15]. Therefore, our analytical approach did not involve a prospectively designed protocol or analysis plan.

We utilized microsimulation models of HPV infection and cervical cancer from 2 independent modeling groups that are part of the Cancer Intervention and Surveillance Modeling Network (CISNET): Harvard University (Harvard) and Cancer Council New South Wales (Policy1-Cervix). Standardized profiles of each model are available at https://cisnet.cancer.gov/cervical/profileshtml. These models estimated the lifetime costs, benefits, and cost-effectiveness of extending HPV vaccination from the earlier guidelines (i.e., up to age 26 years in women; up to age 21 years in men) to vaccinating both women and men up to age 30, 35, 40, or 45 years (i.e., midadult vaccination) in the US. The analysis focused on the nonavalent HPV vaccine (HPV-9) for the new vaccination strategies but incorporated historical vaccination coverage using the quadrivalent vaccine (HPV-4) starting in 2007 for girls and 2010 for boys. We examined the vaccination strategies in the context of recommended cytology-based screening in the US under assumptions of full compliance to screening and management guidelines, which we varied in sensitivity analysis. In addition to cervical disease outcomes, we estimated the vaccine impacts on noncervical HPV-related cancers and genital warts using incidence-based models.

We adopted a healthcare sector perspective, including direct medical costs of disease, vaccination, and cervical cancer screening, irrespective of payer. Health benefits were expressed as quality-adjusted life years (QALYs); costs were expressed in 2018 US dollars. Both costs and benefits were discounted by 3% annually [16]. Using standard methods of cost-effectiveness, we calculated incremental cost-effectiveness ratios (ICERs) and interpreted our results in terms of a recommended threshold range of $50,000 to $200,000 per QALY gained in the US [17]. Reporting was done according to HPV-FRAME standards for models evaluating HPV vaccination in adolescents and adults (S1 HPV-FRAME Checklist) [18].

### Model description

#### HPV and cervical models

**Fig 1:**
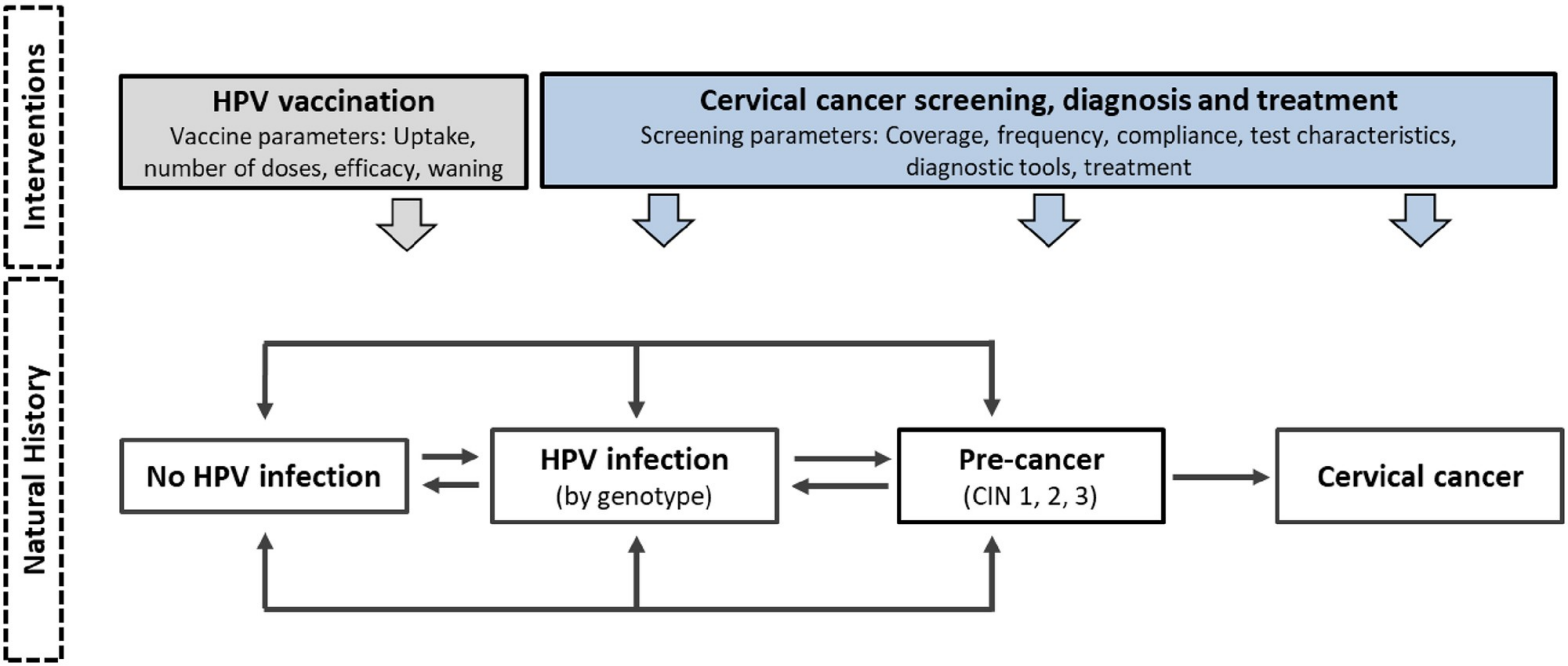
General CISNET-Cervical model schematic. Both CISNET-Cervical models capture distinct phases of the disease process and interventions, including HPV transmission, cervical cancer natural history, HPV vaccination, and cervical screening, diagnosis, and treatment but differ with respect to the number of health states, HPV genotype categorization, histological cancer types, and data sources used to parameterize the baseline model prior to model calibration to the US setting (**Table A in** S1 Text). Disease progression in the model is characterized as a sequence of monthly transitions between health states that are descriptive of each patient’s underlying true health, including infection status, grade of CIN, and stage of cancer. CIN, cervical intraepithelial neoplasia; CISNET, Cancer Intervention and Surveillance Modeling Network; HPV, human papillomavirus.

Both models capture HPV transmission, cervical cancer natural history, HPV vaccination, and cervical screening, diagnosis, and treatment (Fig 1), but differ with respect to the number of health states, HPV genotype categorization, histological cancer types, and data sources used to parameterize the baseline model prior to model calibration to the US setting (**Table A in** S1 Text). HPV transmission in both models is simulated as a function of partnership acquisition and dissolution, by sex, age, and sexual activity level. HPV infection can be transmitted, depending on the number of new partners, partner infection status, probabilities of HPV transmission given an infected partner, and duration of partnership. Individuals who clear an HPV infection develop type-specific natural immunity, which reduces susceptibility to future same-type infection. Cervical carcinogenesis is simulated as a series of transitions through health states that describe underlying true health, including HPV infection, precancer, and invasive cancer. Cancer detection can occur through symptoms or screening. All women face all-cause mortality [19], rates of hysterectomy [20], and excess mortality from cervical cancer [21], which were standardized in both models.

The HPV and cervical models were calibrated to fit to empirical data, including age-specific prevalence of HPV and HPV type distribution observed among females in the US population [22,23]. Model correspondence to age-specific cervical cancer incidence and mortality in the US population was then assessed both in the absence and presence of cervical screening [21]. **Tables B–H in** S1 Text provides details on the model structures, inputs, calibration, and validation.

#### Noncervical HPV-related diseases

We used an incidence-based approach to estimate the impact of HPV vaccination on other HPV-related cancers (i.e., anal, oropharyngeal, vulvar, vaginal, penile) and genital warts; RRP was not included. Data included age- and sex-specific incidence of each cancer and genital warts, proportion of cases attributable to vaccine-targeted HPV types, disease-specific quality of life, average cost per case, and excess mortality (for cancer only) (**Tables I–U in** S1 Text) [24,25]. Although both modeling teams have independently estimated the median natural history dwell time from acquisition of a causal HPV infection to detection of invasive cervical cancer to be 26 years [26,27], for this analysis, we made an assumption favorable to adult vaccination that the dwell time from infection to cancer for each of the noncervical cancers was only 5 years, and no lag time was assumed for vaccine benefits against genital warts. The cancer and warts models were run both without and with the vaccination strategies to generate estimates of QALYs gained and costs averted.

### Analysis

To estimate long-term outcomes associated with extending HPV vaccination to women and men up to age 45 years, we projected the lifetime health and economic consequences for multiple birth cohorts in the US population over years 2019 to 2119.

#### Vaccination strategies

Vaccination coverage was based on data from NIS-TEEN [28–33]. Specifically, the age- and sex-specific annual probabilities of being vaccinated (for individuals not previously vaccinated) were based on the changes in female and male adolescent coverage reported in years 2008, 2009, 2010, 2012, 2014, and 2015 (**Tables V–Y in** S1 Text) [28–33]. In both models, female vaccination up to age 26 years was assumed to start in 2007, and male vaccination up to age 21 years was assumed to start in 2010. In both sexes, vaccination was assumed to start with HPV-4 and transition to HPV-9 from 2015 onwards, based on ACIP recommendations [34]. Likewise, vaccine doses for individuals up to age 14 years was assumed to be 2 doses by the start of the analysis year (2019). We assumed that all vaccinated individuals fully completed their recommended series (either 2 or 3 doses).

Vaccine efficacy was assumed to be 95% over the lifetime against incident HPV infections targeted by the vaccines, irrespective of age. Consistent with the CDC’s assumptions on vaccine costs [24,25], we assumed differential vaccine costs for HPV-9 by age: $410 for 2 doses up to age 14 years, $615 for 3 doses for individuals aged 15 to 18 years, and $675 for 3 doses for individuals aged 19 years and older. In sensitivity analysis, we also evaluated a lower-bound 3-dose vaccine cost of $529 and an upper-bound cost of $704.

#### Cervical cancer screening

We assumed 3-yearly cytology screening in women aged 21 to 65 years, with management of equivocal and abnormal screens according to established guidelines [35–37]. Test sensitivity and specificity values were informed from the published literature and a recent US Preventive Services Task Force analysis (Table 1) [38–42]. Costs associated with screening, diagnosis, and precancer treatment included only direct medical costs and were based on physician and lab fee schedules from the US Centers for Medicare and Medicaid Services [40,41]. Cervical cancer costs were based on a study by Mariotto and colleagues (2011) [42], which reflected phase of cancer care and age. QALYs were estimated by applying health-state or health-event utility weights multiplied by duration in that state (**Table Z in** S1 Text) [43,44].

**Table 1:** Cervical cancer screening parameters*.

Variable		Baseline values
Test characteristics (%)		
Cytology [39]^†^		
Sensitivity (CIN 2+)		0.727
Specificity		0.919
Costs (2018 US dollars) [40,41]^§^		
Screening test^‡^		
Cytology		26
HPV DNA test		46
Office visit		75
Diagnostic follow-up		
Colposcopy/biopsy		146
Lab fee		69
Treatment for CIN 2,3		537
Treatment for cervical cancer [42]^§^		
Initial	<65 years	66,335
	65+ years	55,279
Continuing	All ages	1,744
Terminal	<65 years	144,674
	65+ years	96,449

CIN, cervical intraepithelial neoplasia; HPV, human papillomavirus.

* Common inputs in both Harvard and Policy1-Cervix models.

^†^ Sensitivity (specificity) defined as probability to detect presence (absence) of CIN2+ at a positivity threshold of atypical cells of undetermined significance (AS-CUS).

^‡^ Screening test costs include test and office visit.

^§^ All costs were expressed in 2018 US dollars. Cancer treatment costs are stratified by phase of cancer care and age; initial is first year, terminal is final year (assuming cancer death); continuing is all years between initial and terminal.

We expressed results associated with cervical outcomes only, as well as all HPV-related outcomes (excluding RRP). In the base-case analysis, we assumed that compliance to primary screening and follow-up was as recommended (i.e., perfect). In sensitivity analysis, we used empirical lab-based data from the New Mexico HPV Pap Registry, the only population-based screening registry in the US, to inform current (i.e., imperfect) cervical screening compliance (see **Tables AA–CC in** S1 Text) [45–47]. Other key sensitivity analyses included varying: (1) vaccination costs; (2) incidence rates for noncervical HPV-related diseases; and (3) dwell time from HPV acquisition to invasive cancer for noncervical cancers only.

## Results

### Base-case analysis

**Fig 2:**
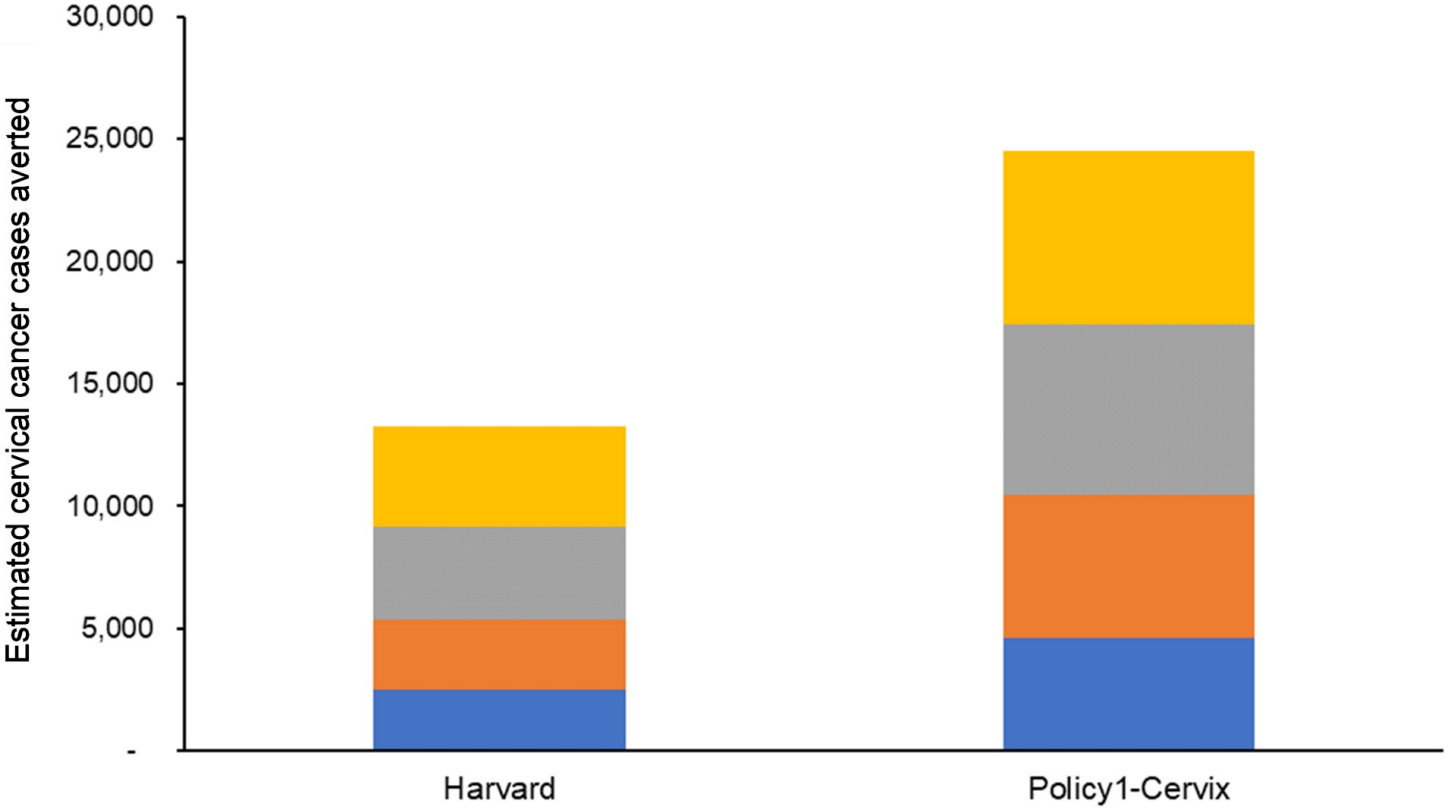
Estimated cervical cancer cases averted. Compared to status quo human papillomavirus (HPV) vaccination, cervical cancer cases averted were projected to continually increase as vaccination extended to women and men ages 30 years (blue), 35 years (orange), 40 years (grey), and 45 years (yellow). Results were calculated based on model projections over the lifetime of 10-year-old girls born in 1969–2009.

Both models projected greater health benefits and higher total costs as the upper age of vaccination increased. Compared to status quo HPV vaccination, cervical cancer cases averted continually increased as vaccination age extended up to 45 years, with an estimated 13,200 (Harvard) to 24,500 (Policy1-Cervix) cases averted over the lifetime of women who belonged to birth cohorts 1969 to 2009 (Fig 2). These results are in the context of 283,300 (Policy1-Cervix) to 324,400 (Harvard) cases that were predicted over the lifetime of these cohorts under the existing status quo adolescent program. When considering only cervical outcomes, both models found that strategies of vaccinating females and males up to ages 30, 35, and 40 years were less effective and less cost-effective than an alternative strategy (i.e., weakly dominated) and that vaccinating up to age 45 years had an ICER ranging from $484,900 to $1,011,400 per QALY gained (Table 2).

**Table 2:** Base-case cost-effectiveness analysis*.

Strategies	Harvard	Policy1-Cervix
Cervical outcomes only^†^		
Vacc to age 30^‡^	dom	dom
Vacc to age 35	dom	dom
Vacc to age 40	dom	dom
Vacc to age 45	$1,011,400	$484,900
All outcomes^§^		
Vacc to age 30^‡^	dom	dom
Vacc to age 35	dom	dom
Vacc to age 40	dom	dom
Vacc to age 45	$440,600	$315,700

* Values represent incremental cost-effectiveness ratios, in terms of costs per quality-adjusted life year (QALY) gained, compared to the next less costly, nondominated strategy; status quo vaccination is the baseline strategy; “dom” indicates dominated strategies that are less costly and less cost-effective (i.e., weakly dominated) than an alternative strategy.

^†^ Only outcomes related to cervical cancer were included.

^‡^ Vaccination to age 30 compared against current vaccination.

^§^ All HPV-related outcomes (cervical, anal, oropharyngeal, vulvar, vaginal, and penile cancers, as well as genital warts) were included.

When considering all HPV-related health outcomes, both models found that the ICER for vaccinating females and males up to age 45 years became more favorable compared to considering cervical-only outcomes; however, the ICER remained high, ranging from $315,700 to $440,600 per QALY gained, and the 3 other strategies remained weakly dominated (i.e., not cost-effective). Including noncervical outcomes was more impactful on the Harvard results than Policy1-Cervix results.

### Sensitivity analysis

We conducted a number of sensitivity analyses (Tables 3 and 4). Both models found that with imperfect cervical screening compliance, vaccinating up to age 40 years was no longer weakly dominated and had an ICER of $177,900 to $245,200 per QALY gained. The ICER for vaccinating up to age 45 years decreased (i.e., became more attractive) to roughly $200,000 per QALY in the case of Policy1-Cervix, and $363,800 per QALY for Harvard.

**Table 3:** Sensitivity analysis: Harvard model (all outcomes)*.

Strategies	Base case (all outcomes)	Imperfect CC screen^†^	Cost per life-year	3-dose HPV vaccine cost = $529	3-dose HPV vaccine cost = $704	20-year lag time for other HPV-related cancers^‡^	40-year lag time for other HPV-related cancers^‡^
Vacc to age 30^||^	dom	dom	dom	dom	dom	dom	dom
Vacc to age 35	dom	dom	dom	dom	dom	dom	dom
Vacc to age 40	dom	$245,200	dom	dom	dom	dom	dom
Vacc to age 45	$440,600	$363,800	$672,800	$340,900	$460,400	$574,500	$651,300

* Values represent incremental cost-effectiveness ratios, in terms of costs per quality-adjusted life year (QALY) gained, compared to the next less costly, nondominated strategy; status quo vaccination is the baseline strategy; “dom” indicates dominated strategies that are less costly and less cost-effective (i.e., weakly dominated) than an alternative strategy. All HPV-related outcomes (except RRP) were included.

^†^ Imperfect cervical cancer screen reflects patterns of screening, diagnosis, and precancer treatment as observed in the New Mexico HPV Pap Registry (see Methods and S1 Text for details).

^‡^ Minimum 20- or 40-year lag time from HPV infection to cancer for non-cervix HPV-related cancers.

^||^ Vaccination to age 30 compared against current vaccination.

**Table 4:** Sensitivity analysis: Policy1-Cervix model (all outcomes)*.

Strategies	Base case (all outcomes)	Imperfect CC screen^†^	Cost per life-year	3-dose HPV vaccine cost = $529	3-dose HPV vaccine cost = $704	40-year lag time for other HPV-related cancers‡	Unfavorable parameters for other HPV-related diseases	Favorable parameters for other HPV-related diseases
Vacc to age 30^‖^	dom	dom	dom	dom	dom	dom	dom	dom
Vacc to age 35	dom	dom	dom	dom	dom	dom	dom	dom
Vacc to age 40	dom	$177,900	dom	dom	dom	dom	dom	dom
Vacc to age 45	$315,700	$199,900	$598,000	$242,800	$330,200	$461,400	$351,400	$270,600

* Values represent incremental cost-effectiveness ratios, in terms of costs per quality-adjusted life year (QALY) gained, compared to the next less costly, nondominated strategy; status quo vaccination is the baseline strategy; “dom” indicates dominated strategies that are less costly and less cost-effective (i.e., weakly dominated) than an alternative strategy. All HPV-related outcomes (except RRP) were included.

^†^ Imperfect cervical cancer screen reflects patterns of screening, diagnosis, and precancer treatment as observed in the New Mexico HPV Pap Registry (see Methods and S1 Text for details).

^‡^ Minimum 40-year lag time from HPV infection to cancer for non-cervix HPV-related cancers.

^‖^ Vaccination to age 30 compared against current vaccination.

When excluding quality of life and considering only life expectancy, the cost-effectiveness of vaccinating up to age 45 years became less favorable, with ratios ranging from $598,000 to $672,800 per life-year saved.

When we lowered vaccine costs ($529 for 3 doses in adults aged 19 years and older), the ICER for vaccinating up to age 45 years became more attractive yet remained above common US willingness-to-pay thresholds; the other midadult vaccination strategies remained weakly dominated in both models. Likewise, the ICER for vaccinating up to age 45 years was less attractive when increasing the 3-dose vaccine cost to $704 for individuals aged 19 years and older.

Both models also evaluated assumptions of extended lag time from HPV vaccination to cancer impact for the noncervical cancers. In both models, the ICERs for vaccinating up to age 45 years increased by over 45% when assuming a 40-year lag between vaccination and cancer impact compared to the base case (i.e., 5-year lag); other vaccination strategies remained weakly dominated.

The Policy1-Cervix model was used for additional sensitivity analysis, simultaneously varying a range of parameters for noncervical HPV-related diseases related to the natural history of these diseases, costs, and the attributable fraction of HPV types. When the most favorable assumptions were simultaneously chosen across these parameters, the findings indicated that vaccination to age 45 years was still unfavorable at $270,600 per QALY, and the other vaccination strategies remained weakly dominated.

## Discussion

This comparative analysis evaluated the cost-effectiveness of increasing the upper age limit for HPV vaccination utilizing 2 well-validated and comprehensive modeling platforms. We found that vaccination to older ages (30, 34, 40, 45 years) was inefficient or associated with unfavorable ICERs in the US context. Sensitivity analysis revealed that assumptions about cervical screening compliance, vaccine costs, and the natural history of noncervical HPV-related cancers could have major impacts on the estimated cost-effectiveness of the vaccination strategies. However, even with the most extreme assumptions, both models almost universally found that the cost-effectiveness of HPV vaccination for adults aged 30 to 45 years was greater than $200,000 per QALY. The only exception was in the scenario assuming imperfect screening compliance, where one model found that vaccinating up to ages 40 and possibly 45 years could be cost-effective if assuming an upper-bound willingness-to-pay threshold of $200,000 per QALY.

It should be noted that the strategies of vaccinating adults aged 30 to 45 years were assessed in the context of 2 prevention strategies already well underway in the US that serve to limit the incremental gains that can be achieved: HPV vaccination in younger cohorts of females and males, which provides some herd immunity benefits for the older unvaccinated cohorts, and cervical screening for women. Additionally, vaccination at older ages can only be effective in those who are susceptible to infection (i.e., those not currently infected but exposed to new infections as they age), and yet the probability of new exposures decreases with age [48]. Furthermore, due to the long median dwell time between infection and cancer development, the benefits of vaccination tend to be accrued several decades into the future and are thus discounted in cost-effectiveness analysis.

A strength of this analysis is that we have employed 2 independent, well-validated models that have been used for a number of evaluations in different settings. Where we vary in inputs, assumptions, or structure reflects the uncertainty in the mechanism of disease or data. This comparative analysis was also done in the context of CDC requesting several independent modeling groups to contribute to the ACIP deliberations. Despite variations across all models, we found that conclusions were qualitatively consistent among those models that were not industry-funded [25,49].

Additionally, this evaluation has been able to harness the extensive work done by the CISNET-Cervical consortium to obtain and standardize empirical data for the US, which were used to inform model inputs. The CISNET-Cervical consortium has focused on detailed modeling of cervical screening recommendations and outcomes; in our sensitivity analysis, both groups considered an imperfect cervical screening scenario, in which both models incorporated detailed empirical data for screening and referral to diagnostic evaluation, and simulated the full recommended range of downstream surveillance and treatment outcomes for screen-positive women. This detail is important because prior evaluations have shown that critical cost savings from vaccination are accrued through the avoidance of referrals for management of screen-detected abnormalities and the range of complex follow-up and surveillance sequalae that such referrals routinely generate [50,51]. On the other hand, cervical screening is a very effective mechanism for secondary prevention, with the absolute effectiveness increasing, particularly in women over the age of 25 to 30 years. In general terms, the impact of secondary prevention of cervical cancer is critical to incorporate, and the CISNET models were able to achieve this in great detail.

Our analysis has several limitations. Where possible, we erred in the direction of making assumptions that were favorable to increasing the age of vaccination, including assuming no delay between the reduction in HPV infections from vaccination and the reduction in genital warts. A similar “incidence-based” approach was used for noncervical cancers by both models, in which we assumed that there could be an impact of HPV vaccination on incidence of noncervical cancers as early as 5 years after vaccination. This minimum lag time was consistent with Chesson and colleagues [24,25] but may be longer in reality. Such assumptions about short dwell times serve to improve the estimated cost-effectiveness of adult vaccination since the health benefits are realized earlier (and therefore not heavily discounted). Unfortunately, evidence to support modeled assumptions about the time between infection and cancer development is very limited. In the case of cervical cancer, where we have leveraged relatively rich data to inform the natural history dwell time from causal HPV infection to invasive cervical cancer, both Harvard and Policy1-Cervix models have estimated the median dwell time to be roughly 26 years [26,27]. Noncervical cancers are typically diagnosed at an older age than cervical cancer (10 to 20 years later) [21]; although cervical screening allows for earlier diagnosis of invasive cancer, this might suggest that the period of time between a causal HPV infection and cancer diagnosis is longer for many noncervical cancers. In sensitivity analysis, both models examined the impact of extending the minimum dwell time to 40 years for noncervical cancers, which, as expected, greatly diminished the cost-effectiveness of vaccination up to age 45 years. Data on the natural history, namely the carcinogenic progression, of the noncervical cancers are uncertain. To assess this uncertainty in more detail, we conducted extensive sensitivity analysis on the prevaccination incidence of the noncervical diseases, the percent of each disease that is attributable to HPV, the survival rate of each cancer, and disutilities associated with each of the HPV-related diseases, and found that vaccination up to ages 30 or older was not cost-effective. However, when more reliable data become available, it will be important to revisit the analyses and update results.

We did not take into consideration potential changes in the future burden of the noncervical cancers (other than through vaccine impact) and instead assumed the current underlying age-specific incidence and mortality rates remained constant over time. To the extent that the incidence rates of these cancers are rising (e.g., oropharyngeal cancers in men), we may be underestimating the overall benefit of HPV vaccination, although it is not clear if the incremental costs and benefits between the different age thresholds for vaccination would be altered.

We also made the favorable assumption that no individuals received less than the recommended dosage. If incomplete dose-course provides reduced or no effectiveness, then the cost-effectiveness of vaccination would be reduced. Furthermore, we made the favorable assumption that HPV-9 was 95% effective at preventing new vaccine-targeted HPV infections in both females and males up to age 45 years and that this protection lasted over their lifetime. There is no trial evidence directly reporting HPV-9 effectiveness in mid-to-older adult females or males. To date, 2 large trials have shown that vaccination with HPV-2 or HPV-4 for females up to age 45 years is somewhat effective at preventing incidence of persistent HPV infection after 4 years of follow-up [8,9]. Additionally there are no trials indicating direct effectiveness of any HPV vaccine in males beyond age 26 years.

The ACIP decision to not recommend catch-up vaccination beyond age 26 years took into consideration the results from our models, which predicted additional health gains when extending HPV vaccination to older ages, but at a disproportionately higher cost. The results from our 2 independent models suggest that HPV vaccination for adult women and men aged 30 to 45 years is unlikely to represent good value for money in the US.

## Supporting information

S1 HPV-FRAME Checklist HPV, human papillomavirus. (DOCX)

S1 Text Model details, input parameters, model calibration, and validation results. (DOCX)

## Abbreviations

ACIP: Advisory Committee on Immunization Practices
CDC: Centers for Disease Control and Prevention
CIN: cervical intraepithelial neoplasia
CISNET: Cancer Intervention and Surveillance Modeling Network
HPV: human papillomavirus
ICER: incremental cost-effectiveness ratio
QALY: quality-adjusted life year
RRP: recurrent respiratory papillomatosis
