# Supplementary material for: Human papillomavirus vaccination for adults aged 30 to 45 years in the United States: A cost-effectiveness analysis
Source: PLoS Med. 2021 Mar 11;18(3):e1003534. doi: 10.1371/journal.pmed.1003534 (PMC7951902; doi:10.1371/journal.pmed.1003534)
Supplement: S1 HPV-FRAME Checklist — (DOCX) [file pmed.1003534.s001.docx]

**S1. HPV-FRAME reporting standard checklist**

The HPV-FRAME checklist is shown below for the core reporting standard and reporting standard for models of HPV vaccination in adolescents and adults [1].

| **Inputs** | **Reported by age? (Y/N)** | | **Report by sex (F/M/Both)?** | | **Comments** |
| --- | --- | --- | --- | --- | --- |
|  | Harvard | Policy1-Cervix | Harvard | Policy1-Cervix |  |
| Target population for intervention | Y | Y | Y | Y | Described in methods section of the main manuscript |
| Sexual behavior | Y | N | Y | Y | Harvard provides detailed tables on age-specific sexual behavior for both sexes in S1 Text. |
|  |  |  |  |  | Policy1-Cervix reports on lifetime numbers of sexual partners and the median age of sexual debut for both sexes in S1 Text. |
| Cohort examined for evaluation/ time horizon | Y (multiple cohorts) | Y (multiple cohorts) | Y (multiple cohorts) | Y (multiple cohorts) | Cost-effectiveness results incorporate multiple birth cohorts as described in the methods section of the main manuscript. The time horizon is also described here. |
| Quality of life assumptions | Yes, for all HPV-related cancers/warts | Yes, for all HPV-related cancers/warts | Yes, for all HPV-related cancers/warts | Yes, for all HPV-related cancers/warts | QALY inputs for all cancers and genital warts, and for cervical screening, are detailed for both Harvard and Policy1-Cervix models in S1 Text. |
| Calibration | Y | Y | Y | Y | Calibration against genital HPV prevalence by age and genotype provided for both Harvard and Policy1-Cervix models in S1 Text. |
|  |  |  |  |  | Calibration against cervical cancer by age in the absence of screening against historical SEER cancer data for cervical cancer is presented for both models in S1 Text. |
| Validation (where possible) | Y | Y | Y | Y | Using observed imperfect screening compliance rates, both models compared model-predicted cervical cancer incidence rates against age-specific cervical cancer incidence in recent years reported by SEER; presented for both models in S1 Text. |
| Costs | Y | Y | Y | Y | Costs for all cancers, cervical screening events and vaccine costs are presented in the main manuscript and in S1 Text. |
| Vaccine coverage | Y | Y | Y | Y | Described in the main manuscript and in S1 Text. |
| Vaccine uptake | Y | Y | Y | Y | Detailed uptake by age, sex, and year described in S1 Text. |
| Vaccine efficacy | Y | Y | Y | Y | Efficacy against vaccine-targeted HPV at any site is assumed to be 95% for both women and men, at any age, as described in the methods section in main manuscript. |
| Vaccine cross-protection | Y | Y | N/A | N/A | Efficacy is assumed to apply against vaccine-included types only, as described in the main manuscript. |
| Duration vaccine protection and waning | Y | Y | Y | Y | Duration is assumed to be lifelong against HPV at any site, for both sexes and any age, as described in the methods section in the main manuscript. |
| Vaccine and delivery costs | Y | Y | Y | Y | Vaccine cost is described in the methods section of the main manuscript and does not differ by sex. |
| Pre-vaccination disease burden (including population attributable fractions for HPV) | Y | Y | N/A | N/A | Model-predicted pre-vaccination genital HPV prevalence by age and genotype is described in S1 Text and compared to real-world observed data. An incidence-based approach was used for all other HPV-related cancers and genital warts in both men and women, and prevalence of cancers and genital warts is described in detail for both sexes and by age, in S1 Text. |
| Duration of natural immunity | Y | N | Y | N | Duration of natural immunity by age and sex are described for the models in S1 Text. |
| Natural history parameters, specifically for older individuals | Y | N | Y | N | The Harvard model has reported progression status by age, time since infection and HPV type.  Re-activation not modelled by either group; infections in older women are assumed to be new infections (favorable assumption for minimizing cost-effectiveness of adult vaccination). |
| Screening impact for vaccinated individuals | N/A | N/A | N/A | N/A | Screening not assumed to change, and vaccination independent of screening attendance. |
| **Outputs** | **Reported by age? (Y/N)** | | **Report by sex (F/M/Both)?** | | **Comments** |
| Cancer incidence, mortality, life years, QALYs/DALYs (as appropriate) | Y | Y | Y | Y | Both models report on cervical cancer incidence and mortality as calibration and validation.  All other HPV-related cancers, and genital warts, used an incidence-based approach; data used for these cancers by age and for both men and women are reported in S1 Text. |
| HPV prevalence, pre-intervention | Y | Y | N/A | N/A | Both models report on female genital HPV prevalence pre-intervention as a calibration target against observed data, by age and HPV genotype. An incidence-based approach was used for all other HPV-related cancers and genital warts in both men and women, and HPV prevalence at these sites is not applicable for this approach. |
| CIN2/3 detected | N | N | N | N | CIN2/3 was not reported as a separate output but was captured in QALY calculations and cost calculations. |
| Sensitivity analysis on key inputs | Yes (N/A for age-based presentation) | Yes (N/A for age-based presentation) | N/A | N/A | Sensitivity analysis on a range of key parameters was performed and presented in the main manuscript. |
| Incremental cost-effectiveness ratios and costs saved | Yes (N/A for age-based presentation) | Yes (N/A for age-based presentation) | N/A | N/A | ICERs for base-case and sensitivity analysis are presented. |
| Absolute reductions in HPV infections, and/or warts, post-vaccination | N | N | N | N | Not reported explicitly, although both models capture reductions in infection and disease, and these reductions are captured in QALY estimates for cost-effectiveness. |
| Absolute reductions in invasive cancer (cervical and other HPV cancers, as relevant) post-vaccination | N | N | N | N | Not reported explicitly, although both models capture reductions in disease and these reductions are captured in QALY estimates for cost-effectiveness. |

**Reference:**

1. Canfell K, Kim JJ, Kulasingam S, Berkhof J, Barnabas R, Bogaards JA, et al. HPV-FRAME: A consensus statement and quality framework for modelled evaluations of HPV-related cancer control. *Papillomavirus Res*. 2019;8:100184. doi:10.1016/j.pvr.2019.100184.
