# Supplementary material for: Human papillomavirus vaccination for adults aged 30 to 45 years in the United States: A cost-effectiveness analysis
Source: PLoS Med. 2021 Mar 11;18(3):e1003534. doi: 10.1371/journal.pmed.1003534 (PMC7951902; doi:10.1371/journal.pmed.1003534)
Supplement: S1 Text — (DOCX) [file pmed.1003534.s002.docx]

**S1. Human papillomavirus vaccination for adults aged 30 to 45 years in the United States: A cost-effectiveness analysis**

Jane J. Kim; Kate T. Simms; James Killen; Megan A. Smith; Emily A. Burger; Stephen Sy; Catherine Regan; Karen Canfell

1. Model descriptions 3

1.1 CISNET overview 3

1.2 Harvard models 3

1.2.1 HPV transmission model 4

1.2.2 Cervical cancer natural history model 5

1.3 Policy1-Cervix model 6

2. Model calibration and validation 8

3. Non-cervical HPV-related diseases 9

3.1 Harvard model 10

3.2 Policy1-Cervix model 10

4. Analysis and strategies 11

4.1 HPV vaccination 12

4.2 Cervical cancer screening 14

References 16

**Tables**

Table A. Comparison of main model attributes

Table B. Annual number of total partnerships by age and sexual activity category, males

Table C. Annual number of total partnerships by age and sexual activity category, females

Table D. Average partnership duration in months (95% CI) by age and sexual activity category

Table E. Assortativeness

Table F. HPV clearance by time since infection

Table G. Harvard calibrated parameter set for dynamic model

Table H. Harvard calibrated parameter set for stochastic model

Table I. Annual vulvar cancer incidence rates (per 100,000)

Table J. Annual vaginal cancer incidence rates (per 100,000)

Table K. Annual penile cancer incidence rates (per 100,000)

Table L. Annual anal cancer incidence rates, males (per 100,000)

Table M. Annual anal cancer incidence rates, females (per 100,000)

Table N. Annual oropharyngeal cancer incidence rates, males (per 100,000)

Table O. Annual oropharyngeal cancer incidence rates, females (per 100,000)

Table P. Annual genital warts incidence rates, males (per 100,000)

Table Q. Annual genital warts incidence rates, females (per 100,000)

Table R. Percent of cervical, vulvar, vaginal, penile, anal and oropharyngeal cancers attributable to HPV types

Table S. Base case estimates and ranges of the cost per case of non-cervical HPV-related diseases (2018 U.S. dollars)

Table T. Quality of life detriments for treatment for non-cervical HPV-associated diseases

Table U. Relative 5-year cancer survival probabilities

Table V. Harvard vaccine uptake rates in females (current “status quo” scenario)

Table W. Harvard vaccine uptake rates in males (current “status quo” scenario)

Table X. Policy1-Cervix vaccine uptake rates in females (current “status quo” scenario)

Table Y. Policy1-Cervix vaccine uptake rates in males (current “status quo” scenario)

Table Z. Utilities for cervical screening and cancer

Table AA. Proportion (%) of population screening at different frequencies

Table BB. Proportion of women who received colposcopy/biopsy, by cytology result

Table CC. Proportion of women who received precancer treatment, by histology result

Table DD. Incremental costs and QALYs associated with HPV vaccination strategies (base-case cost-effectiveness analysis)

**Figures**

Figure A. Schematic of Harvard HPV transmission model

Figure B. Schematic of Harvard cervical cancer natural history model

Figure C. Schematic of Policy1-Cervix model

Figure D. Calibration results: Model fit to HPV prevalence

Figure E. Calibration results: Model fit to HPV type distribution

Figure F. Validation results: Model fit to cervical cancer incidence rates, pre-screening

Figure G. Validation results: Model fit to cervical cancer mortality rates, pre-screening

Figure H. Validation results: Cervical cancer incidence rates by age under assumptions of imperfect screening and follow-up compliance, compared to SEER data

Figure I. Validation results: Cervical cancer mortality rates by age under assumptions of imperfect screening and follow-up compliance, compared to SEER data

**1. MODEL DESCRIPTIONS**

1.1 CISNET overview

The cervical cancer working group of the Cancer Intervention and Surveillance Modeling Network (CISNET-Cervical) comprises five independent modeling groups that have well-established mathematical models of HPV and cervical carcinogenesis. These models can be used to conduct standardized, comparative analyses to address concerns regarding model transparency and help guide public health research and priorities (<https://cisnet.cancer.gov/cervical/profiles>).

We utilized HPV and cervical cancer microsimulation models from two CISNET-cervical sites: Harvard University (Harvard) and Cancer Council New South Wales (Policy1-Cervix). Both models have been applied extensively to inform guidelines and decision-making regarding HPV vaccination and cervical cancer screening policies throughout the world; they also represent two of three models that formed the Cervical Cancer Elimination Modelling Consortium, which directly informed the World Health Organization’s strategic plan for global cervical cancer elimination.

Both CISNET-Cervical models capture distinct phases of the disease process and interventions, including HPV transmission, cervical cancer natural history, HPV vaccination, and cervical screening, diagnosis, and treatment but differ with respect to the type and number of health states, HPV genotypes included, histological cancer types, and data sources used to parameterize the baseline model prior to model calibration) to the US setting (Table A).

1.2 Harvard models

The Harvard model includes (1) an individual-based dynamic (i.e., agent-based) model to simulate sexual transmission of the seven high-risk HPV genotypes targeted by HPV-9 (HPV-16/18/31/33/45/52/58, each modeled independently) between males and females, and (2) an individual-based static (i.e., stochastic) model to simulate cervical carcinogenesis and screening associated with all HPV genotypes. These models have been calibrated, validated, and applied extensively to inform HPV vaccination and cervical screening policy decisions in the United States and globally. Previous versions of the models were used to inform CDC recommendations on catch-up vaccination in women [1], routine vaccination of boys [2], and targeted vaccination of HIV-positive men who have sex with men [3]. The current versions of the models were recently used to directly inform U.S. Preventive Services Task Force national guidelines on primary HPV testing [4], and to evaluate potential cost-effective changes to cervical screening policy in HPV-vaccinated women [5]. In low- and middle-income countries, they have been used to evaluate the impact and cost-effectiveness of single-dose HPV vaccination [6], adoption of HPV DNA testing and triage strategies [7-13], optimal screening in HIV-positive women [14], and cervical cancer elimination globally [15-17].

1.2.1 HPV transmission model

The individual, agent-based dynamic model simulates heterosexual partnership acquisition and dissolution, and independent transmission of seven HPV genotypes (HPV-16, -18, -31, -33, -45, -52, -58) (Figure A). Individuals are stratified by sex, age, and sexual activity category (SAC; four categories: none (0), low (1), medium (2), high (3)), which govern initial sexual mixing in the population. Each month, individuals in the model cycle through four steps: (1) sexual mixing, (2) HPV infection, (3) HPV clearance and natural immunity, (4) aging, births, and deaths. For each male in the population, the annual number of partnerships (P) is assigned as a function of age and SAC (Table B) [18]. Partnership assessment occurs at the start of each year of each male’s life, and any new partnerships are formed throughout the course of the upcoming year. For males who are missing one or more female partner(s), a new partnership is formed based on a woman’s age and SAC (Table C) [18], with the duration (D) of each partnership randomly drawn from age- and SAC-specific normal distributions (Table D) [18]. Mixing is assumed to be slightly assortative with respect to age and SAC (Table E) [18]. HPV transmission may occur between discordant partners. Sex-specific clearance of an HPV infection allows HPV natural immunity to increase exponentially with each acquisition and clearance of the same HPV genotype (Table F) [19,20]. Individuals are eligible to form another partnership, irrespective of ongoing partnerships (i.e., concurrency).

The model was calibrated to (i.e., calibration targets) age- and genotype-specific HPV prevalence [21] by varying natural immunity and the monthly partnership transmission probability. The calibrated model parameter values used in this analysis are summarized in Table G**.**

1.2.2 Cervical cancer natural history model

The individual-based stochastic model simulates HPV-induced cervical carcinogenesis associated with all HPV types. Disease progression in the model is characterized as a sequence of monthly transitions between health states. Health states in the model, descriptive of each patient’s underlying true health, include infection status, grade of cervical intraepithelial neoplasia (CIN), and stage of cancer (Figure B). HPV types are stratified as HPV-16; -18; -31; -33; -45; -52; -58; other high-risk infections pooled; and low-risk infections pooled. Health states reflecting invasive cancer include both detected and undetected cancer; a cancer is considered detected when either symptoms occur that lead to a correct diagnosis or a previously undiagnosed malignancy is detected by screening. The probabilities governing these transitions depend on age; HPV type; duration of HPV infection; type-specific natural immunity; as well as a woman’s history of prior infection; and previously treated CIN. All women are at risk for death from other causes. Women without cancer or with undetected cancer are additionally subjected to age-specific risks of hysterectomy, after which all HPV-related disease progression stops, and these women are only subject to the risk of death from other causes.

The generated HPV age- and type-specific HPV incidence reductions over time from the dynamic model serve as inputs to the stochastic model, which is used to capture the direct and indirect effects of HPV vaccination strategies in the context of cervical cancer screening in multiple birth cohorts over time.

The Harvard models were calibrated to empirical data using a likelihood-based approach. Calibrated parameters included HPV incidence (by age and genotype), CIN progression and regression, and HPV natural immunity following type-specific HPV infection and clearance. Baseline values for each of the uncertain parameters were randomly selected from a pre-determined plausible range, creating a unique natural history parameter set. Goodness of fit was ascertained by calculating the likelihood of model-projected outcomes from each parameter set against corresponding calibration targets. The calibrated model parameter values used in this analysis are summarized in Table H [19,22-31].

1.3 Policy1-Cervix

Policy1-Cervix is a dynamic model of HPV transmission, HPV vaccination, cervical precancer, cancer survival, screening, diagnosis and treatment (Figure C). Policy1-Cervix has been used to estimate the timeframe until cervical cancer elimination in Australia [32], globally [33], in 78 low- and lower-middle income countries [15,16], and in the United States [17]. Policy1-Cervix has also been used for many policy evaluations performed on behalf of government agencies. For example, it was used to evaluate changes to the cervical cancer screening interval in Australia and the United Kingdom [34,35], the role of alternative technologies for screening in Australia, New Zealand and England [35-38], the cost-effectiveness of alternative screening strategies and combined screening and vaccination approaches in China [39,40], and the impact of HPV vaccine hesitancy in Japan [41]. The platform was used to perform the effectiveness modeling and economic evaluation of cervical screening for both unvaccinated cohorts and cohorts offered vaccination, as part of evaluation of the role of primary HPV screening in Australia [42,43], New Zealand [44] and England [45]; this work directly supported a transition to primary HPV screening in both Australia and New Zealand. The model has also been used to evaluate the impact of the HPV-9 on cervical screening in four developed countries [46] and to assess the cost-effectiveness of the HPV-9 in Australia [47]. Policy1-Cervix has been extensively calibrated and validated to data from several countries [42,48-50]. For example, predictions from the dynamic HPV transmission and vaccination model have been validated against observed declines in HPV prevalence in women aged 18-24 after the introduction of the quadrivalent vaccine in Australia [48]. Model predictions of age-specific cervical cancer incidence and mortality, rates of histologically confirmed high-grade lesions per 1,000 women screened, and screening adherence rates have been previously validated against national data from Australia, England and New Zealand after taking into account local screening behavior obtained via analysis of screening registry data [42,49]. It has also recently been used to predict the short-term transitional impacts of changing to longer-interval HPV testing and the combined impact of these screening changes and HPV vaccination in Australia to 2035 [51,52].

The model simulates HPV infection which can persist and/or progress to cervical intraepithelial neoplasia grades I, II and III (CIN1, CIN2, CIN3); CIN 3 can then progress to invasive cervical cancer. Progression and regression rates between states are modelled separately for types HPV 16, HPV 18, pooled other nonavalent-included high-risk types (31/33/45/52/58), and pooled other non-nonavalent-included high risk types. To capture the impact of HPV vaccination, we used a general compartmental dynamic transmission model. The dynamic transmission model stratified the population by sex, 5-year age group, and 4 sexual behavior classes, each with varying levels of activity, defined by the annual number of new sexual partners. More details on the parameter assumptions for the dynamic model can be found in previous publications [53]. The dynamic transmission model assumes a median age of sexual debut of 16-17 for females and males, and a median lifetime number of sexual partners of 4 in females and 7 in males, which are consistent with reported sexual behavior data from United States [18]. This component interfaces with the microsimulation implementation of the natural history model and the Screening and Treatment model.

The natural history component of the model (i.e. the states specifying progression/regression to CIN states and development of cancer, following infection with a specific HPV type) was assumed to be unchanged between settings; however, we calibrated the HPV incidence curve to match data representative of the United States and, as described above, we incorporated empirical data on cervical screening uptake in one scenario simulated (rather than assuming perfect adherence to screening and management recommendations).

**2. MODEL CALIBRATION AND VALIDATION**

The sources for calibration target data were selected on the basis of representativeness of the general population, sampling methods, and sample size. All data were from populations prior to HPV vaccination. Age-specific prevalence of HPV infections was based on data from the New Mexico HPV Pap Registry (NMHPVPR), the only statewide screening registry in the United States [21]. HPV type distribution in cases of CIN and cancer were also included as calibration target data. For CIN 2 and CIN 3, HPV type distribution was based on data from the NMHPVPR [54]; for cancer, HPV type distribution in cancer was based on a recent study by the U.S. Centers for Disease Control and Prevention (CDC) using tissue samples from US population-based cancer registries [55]. Model fit to calibration targets are displayed in Figures D-E.

We assessed the fit of the model in the absence of screening to rates of cervical cancer incidence and mortality from the Connecticut Tumor Registry (CTR) before screening was widely available (1950-1969) [56] (Figures F-G).

When we simulated screening practice assuming variable cytology testing rates and imperfect compliance to diagnostic testing and precancer treatment, according to the New Mexico HPV Pap Registry [57-59], our models predicted rates of cervical cancer incidence and mortality that are consistent with those reported more recently in SEER (2000-2013) [31] (Figures H-I).

**3. NON-CERVICAL HPV-RELATED DISEASES**

Additional disease outcomes considered for the current evaluation were anal, oropharyngeal, vulvar, vaginal, and penile cancers, as well as genital warts; simulated impact of HPV vaccination on RRP (juvenile or adult onset) was not included. Inputs for non-cervical HPV-related diseases were standardized based on data from Chesson et al. [60,61], including age- and sex-specific annual incidence rates for vulvar, vaginal, penile, anal, and oropharyngeal cancers, as well as genital warts (**Tables I-Q**); proportion of cases attributable to HPV types (**Table R**); cost per cancer and genital warts case (**Table S**); quality of life weights (i.e., utilities) (**Table T**); and relative 5-year cancer survival probabilities (**Table U**). The cancer and warts models were run both without and with the vaccination strategies to generate estimates of QALYs gained and costs averted with each vaccination strategy by age and time for the entire U.S. population over the lifetimes of birth cohorts affected over the years 2019-2119. Costs were expressed in 2018 U.S. dollars. Both QALYs and costs were discounted at a rate of 3% per year, starting in year 2019.

3.1 Harvard

For the non-cervical HPV-related cancers, the agent-based dynamic model was used to estimate the pooled incidence reductions of the seven high-risk types associated with each vaccination strategy by age, birth cohort, and time. These reductions were adjusted to reflect the decreasing probability that cancer-causing HPV infections are acquired as individuals age, based on a previous model-based analysis of age of causal HPV infections [62] and were then applied to each of the age-specific cancer incidence rates and attributable HPV-9 proportions. Because of the long dwell time between HPV infection and cancer incidence, we imposed a 5-year lag time before vaccine impact was applied based on model-estimated median age of acquisition of causal HPV infection for cervical cancer [62] and median age of development of each of the non-cervical cancers [31].

For genital warts, HPV-6/11 infections were not explicitly modeled, but the pooled incidence reductions of the seven high-risk types were used as a surrogate for HPV-6/11; no lag time was assumed for vaccine benefits against genital warts.

3.2 Policy1-Cervix

The impact of HPV vaccination on genital warts was calculated by multiplying the incidence of genital warts in the absence of HPV vaccination, by the vaccine-preventable fraction of genital warts and by the age- and calendar-year specific relative reduction in HPV-6/11 infections expected due to vaccination with either HPV-4 or HPV-9. As HPV-6/11 are not explicitly modeled, the relative reduction in HPV-18 infections predicted by the transmission model was used as a surrogate for the relative reduction in HPV-6/11 infections, as previous analyses have estimated that the reduction in HPV-6/11 is more similar to that in HPV-18 than in HPV-16 [63]. It was assumed that the reduction in infections would be translated to a commensurate reduction in genital warts in the same year, due to the relatively short period between infection incidence and clinical presentation of anogenital warts [64].

The impact on included non-cervical cancers (oropharyngeal, anal, vulvar, vaginal and penile) was calculated in an analogous way to the reduction in genital warts, which in each case took into account the fraction of each cancer type attributable to HPV types prevented by HPV-4 and HPV-9. To account for the longer period of time between HPV infection and diagnosis of these other cancers (i.e., the longer dwell times associated with development of cancers vs. warts), we calculated the percent reduction in cumulative risk of HPV infection (rather than the age-specific incidence) by a given age. Additionally, a minimum time period was set between the timing of vaccination and the earliest impact on non-cervical HPV-related cancers. This approach is analogous to the one used by Chesson et al. [60,61], and the same minimum lag period of five years was used.

**4. ANALYSIS AND STRATEGIES**

To estimate long-term outcomes associated with extending HPV vaccination to mid-adult women and men, we projected the lifetime health and economic consequences for multiple birth cohorts in the U.S. population from years 2019-2119. The Harvard analysis captured the lifetime effects and costs for the entire U.S. population over the full period. The Policy1-Cervix model simulated birth cohorts born in 1969-2009 and assumed that outcomes for birth cohorts before 1969 were equivalent to those for the 1969 birth cohort; similarly, birth cohorts after 2009 were assumed to have equivalent outcomes to the 2009 birth cohort. The assumption that birth cohorts from 2010 onwards have equivalent outcomes to birth cohorts in 2009 is favorable with respect to adult vaccination as the herd effects from the existing adolescent vaccination program become larger for later cohorts, and the differences in costs and benefits between strategies diminish over time. The underlying population structure by age from years 2019 onward was obtained from the United Nations World Population Prospects: The 2017 Revision (United Nations, Department of Economic and Social Affairs, Population Division (2017). World Population Prospects: The 2017 Revision, DVD Edition).

Using standard methods of cost-effectiveness, we calculated incremental cost-effectiveness ratios (ICERs) and interpreted our results in terms of a recommended threshold range of $50,000 to $200,000 per QALY gained in the United States [65]. After eliminating strategies that were more costly and less effective (i.e., strongly dominated) or less costly and less cost-effective (i.e., weakly dominated) than an alternative strategy, incremental cost-effectiveness ratios were calculated as the additional cost divided by the additional health benefit of one strategy compared to the next-less-costly strategy. Although there is no consensus on a ‘cut-point’ for good value for resources, we interpreted our results in terms of a recommended threshold range of $50,000 to $200,000 per QALY gained in the United States [65].

4.1 HPV vaccination

Vaccination coverage was based on data from NIS-TEEN [66-71]. Specifically, the age- and sex-specific annual probabilities of being vaccinated (for individuals not already vaccinated) was based on the changes in female and male adolescent coverage reported in NIS-TEEN interviews undertaken in 2008, 2009, 2010, 2012, 2014 and 2015 [66-71]. In both models, female vaccination up to age 26 years was assumed to start in 2007, and male vaccination up to age 21 was assumed to start in 2010. In both sexes, vaccination was assumed to start with HPV-4 and transition to using HPV-9 from 2015 onwards, based on the updated ACIP recommendations [72]. Likewise, vaccine doses for individuals up to age 14 years was assumed to be two doses by the start of the analysis year (2019). We assumed that all vaccinated individuals fully completed their recommended series (either two or three doses).

In the Harvard model, coverage in females started at age 12 in 2007 and continued in subsequent years, based on uptake achieved by age 13 in NIS-TEEN years 2014-15 (i.e., 29.5%) (**Table V**) [66-71]. Coverage for 13- to 18-year-old females in 2007 was based on age-specific uptake in years 2007-08 (coverage for 18-year-old females was assumed to be the same as for 17-year-olds). In subsequent years, annual uptake rates were assumed to be 12.9% in females aged 13-18 years, based on the observed increase in uptake in this age range between 2014 and 2015, and 2.6% (20% of the rate of those aged 13-18 years) in females aged 19-26 years (status quo scenario) and up to age 45 years (new strategies); these uptake rates were consistent with those in other ACIP analyses [60,61]. In males, coverage started at age 12 in 2010, based on uptake achieved by age 13 in years 2014-15 (i.e., 24.9%) (**Table W**) [68-71]. Similar to female coverage, coverage for 13- to 18-year-old males in 2010 was based on age-specific uptake in years 2010-11. In subsequent years, annual uptake rates were assumed to be 9.7% in males aged 13-18 years, based on the observed increase in uptake in this age range between 2014 and 2015, and 1.9% (20% of the rate of those aged 13-18 years) in males aged 19 and older.

In the Policy1-Cervix model, age- and sex-specific uptake for years 2007-2015 was also based on the NIS-TEEN data (**Tables X-Y**) [66-71]. Specifically, it was set so that the sex-specific cumulative uptake would match coverage reported at each single year of age from 13 through 17 years, in NIS-TEEN interviews undertaken in 2008, 2009, 2010, 2012, 2014 and 2015. For initial years of vaccination, cumulative coverage reported at age 13 was assumed to be achieved specifically at age 13 (rather than at age 12 years or younger). From 2016 onwards, uptake was assumed to be stable, and the same as that used in the Harvard model and other ACIP analysis [60,61]; uptake was set at 29.5% and 24.9% in 12-year-old females and males, respectively, based on coverage reported at age 13 in NIS-TEEN in 2015 [71]. Among those aged 13-18 years, uptake was 12.9% in females and 9.7% in males, based on the observed increase in uptake between 2014 and 2015 (i.e., average of the sex- and age-specific increases in those aged 13 to 16 years in 2014). Uptake among those aged 18 years was assumed to be the same as in those aged 17 years. As with the Harvard model and other ACIP analyses [60,61], uptake in females aged 19-26 years (status quo scenario) and older (new vaccination scenarios) was assumed to be 2.6% (20% of the rate in females aged 13-18 years). Similarly, uptake in males aged 19-21 years (status quo) and older (new scenarios) was assumed to be 1.9% (20% of the rate in males aged 13-18 years).

4.2 Cervical cancer screening

Screening assumptions in the model can vary by screening start age, stop age, interval between negative screens, coverage, triage testing, and compliance to recommended follow-up. Management of screen-positive women can vary by age, follow-up test, time to follow-up test(s), and number of negative follow-up tests required to return to routine screening. In this analysis, we evaluated mid-adult vaccination in the context of 3-yearly cytology testing from ages 21-65 years, a recommended cervical cancer screening strategy for U.S. women since 2012 [73,74]. Management of women with equivocal or abnormal screening results followed established guidelines [75]; in particular, women with atypical squamous cell of undetermined significance (ASC-US) received reflex HPV testing, while those with low-grade or high-grade squamous intraepithelial lesions (LSIL or HSIL) or atypical squamous cells indicating high-grade (ASC-H) were referred to diagnostic colposcopy/biopsy. Women with histologically-confirmed high-grade lesions (i.e., CIN2+) were referred for excisional treatment.

Both models captured decreased quality of life associated with detected cervical cancer based on stage at diagnosis (**Table Z**) [76-78]. In addition, Policy1-Cervix reflected disutility associated with investigative testing (without treatment) and disutility associated with precancer treatment. Neither model applied background age-specific utility weights for those without disease.

In the base-case analysis, we assumed perfect compliance to screening and follow-up; however, in sensitivity analysis, we also examined a scenario of imperfect compliance based on data from the New Mexico HPV Pap Registry (**Tables AA-CC**) [57-59].

**REFERENCES**

1. Kim JJ, Goldie SJ. Health and economic implications of HPV vaccination in the United States. N Engl J Med. 2008;359(8):821-32. PMID: 18716299.

2. Kim JJ, Goldie SJ. Cost effectiveness analysis of including boys in a human papillomavirus vaccination programme in the United States. BMJ. 2009;339:b3884. PMID: 19815582.

3. Kim JJ. Targeted human papillomavirus vaccination of men who have sex with men in the USA: a cost-effectiveness modelling analysis. Lancet Infect Dis. 2010;10(12):845-52. Epub 2010/11/06. doi: 10.1016/S1473-3099(10)70219-X. PMID: 21051295; PMCID: PMC3982926.

4. Kim JJ, Burger EA, Regan C, Sy S. Screening for Cervical Cancer in Primary Care: A Decision Analysis for the US Preventive Services Task Force. JAMA. 2018;320(7):706-14. Epub 2018/08/25. doi: 10.1001/jama.2017.19872. PMID: 30140882.

5. Kim JJ, Burger EA, Sy S, Campos NG. Optimal Cervical Cancer Screening in Women Vaccinated Against Human Papillomavirus. J Natl Cancer Inst. 2017;109(2):djw216. doi: 10.1093/jnci/djw216. Epub 2016/10/19. doi: 10.1093/jnci/djw216. PMID: 27754955; PMCID: PMC5068562.

6. Burger EA, Campos NG, Sy S, Regan C, Kim JJ. Health and economic benefits of single-dose HPV vaccination in a Gavi-eligible country. Vaccine. 2018;36(32 Pt A):4823-9. Epub 2018/05/29. doi: 10.1016/j.vaccine.2018.04.061. PMID: 29807710; PMCID: PMC6066173.

7. Campos NG, Jeronimo J, Tsu V, Castle PE, Mvundura M, Kim JJ. The Cost-Effectiveness of Visual Triage of Human Papillomavirus-Positive Women in Three Low- and Middle-Income Countries. Cancer Epidemiol Biomarkers Prev. 2017;26(10):1500-10. Epub 2017/07/16. doi: 10.1158/1055-9965.EPI-16-0787. PMID: 28710075.

8. Campos NG, Maza M, Alfaro K, Gage JC, Castle PE, Felix JC, et al. The comparative and cost-effectiveness of HPV-based cervical cancer screening algorithms in El Salvador. Int J Cancer. 2015;137(4):893-902. Epub 2015/02/03. doi: 10.1002/ijc.29438. PMID: 25639903.

9. Campos NG, Mvundura M, Jeronimo J, Holme F, Vodicka E, Kim JJ. Cost-effectiveness of HPV-based cervical cancer screening in the public health system in Nicaragua. BMJ open. 2017;7(6):e015048. Epub 2017/06/18. doi: 10.1136/bmjopen-2016-015048. PMID: 28619772; PMCID: PMC5623348.

10. Campos NG, Tsu V, Jeronimo J, Mvundura M, Kim JJ. Estimating the value of point-of-care HPV testing in three low- and middle-income countries: a modeling study. BMC cancer. 2017;17(1):791. Epub 2017/11/28. doi: 10.1186/s12885-017-3786-3. PMID: 29178896; PMCID: PMC5702206.

11. Campos NG, Tsu V, Jeronimo J, Njama-Meya D, Mvundura M, Kim JJ. Cost-effectiveness of an HPV self-collection campaign in Uganda: comparing models for delivery of cervical cancer screening in a low-income setting. Health Policy Plan. 2017;32(10):1491. Epub 2017/10/04. doi: 10.1093/heapol/czx076. PMID: 28973511; PMCID: PMC5886202.

12. Campos NG, Tsu V, Jeronimo J, Regan C, Resch S, Clark A, et al. Health impact of delayed implementation of cervical cancer screening programs in India: A modeling analysis. Int J Cancer. 2019;144(4):687-96. Epub 2018/08/23. doi: 10.1002/ijc.31823. PMID: 30132850.

13. Mezei AK, Pedersen HN, Sy S, Regan C, Mitchell-Foster SM, Byamugisha J, et al. Community-based HPV self-collection versus visual inspection with acetic acid in Uganda: a cost-effectiveness analysis of the ASPIRE trial. BMJ Open. 2018;8(6):e020484. Epub 2018/06/14. doi: 10.1136/bmjopen-2017-020484. PMID: 29895648; PMCID: PMC6009460.

14. Campos NG, Lince-Deroche N, Chibwesha CJ, Firnhaber C, Smith JS, Michelow P, et al. Cost-Effectiveness of Cervical Cancer Screening in Women Living With HIV in South Africa: A Mathematical Modeling Study. J Acquir Immune Defic Syndr. 2018;79(2):195-205. Epub 2018/06/20. doi: 10.1097/QAI.0000000000001778. PMID: 29916959; PMCID: PMC6143200.

15. Brisson M, Kim JJ, Canfell K, Drolet M, Gingras G, Burger EA, et al. Impact of HPV vaccination and cervical screening on cervical cancer elimination: a comparative modelling analysis in 78 low-income and lower-middle-income countries. Lancet. 2020;395(10224):575-90. Epub 2020/02/03. doi: 10.1016/S0140-6736(20)30068-4. PMID: 32007141; PMCID: PMC7043009.

16. Canfell K, Kim JJ, Brisson M, Keane A, Simms KT, Caruana M, et al. Mortality impact of achieving WHO cervical cancer elimination targets: a comparative modelling analysis in 78 low-income and lower-middle-income countries. Lancet. 2020;395(10224):591-603. Epub 2020/02/03. doi: 10.1016/S0140-6736(20)30157-4. PMID: 32007142; PMCID: PMC7043006.

17. Burger EA, Smith MA, Killen J, Sy S, Simms KT, Canfell K, et al. Projected time to elimination of cervical cancer in the USA: a comparative modelling study. Lancet Public Health. 2020;5(4):e213-e22. Epub 2020/02/15. doi: 10.1016/S2468-2667(20)30006-2. PMID: 32057315.

18. National Center for Health Statistics. National Survey of Family Growth. <https://www.cdc.gov/nchs/nsfg/nsfg_2011_2013_puf.htm> (last accessed January 3, 2021).

19. Herrero R, Hildesheim A, Rodriguez AC, Wacholder S, Bratti C, Solomon D, et al. Rationale and design of a community-based double-blind randomized clinical trial of an HPV 16 and 18 vaccine in Guanacaste, Costa Rica. Vaccine. 2008;26(37):4795-808. Epub 2008/07/22. doi: 10.1016/j.vaccine.2008.07.002. PMID: 18640170; PMCID: PMC2652516.

20. Giuliano AR, Palefsky JM, Goldstone S, Moreira ED, Jr., Penny ME, Aranda C, et al. Efficacy of quadrivalent HPV vaccine against HPV Infection and disease in males. N Engl J Med. 2011;364(5):401-11. Epub 2011/02/04. doi: 10.1056/NEJMoa0909537. PMID: 21288094.

21. Wheeler CM, Hunt WC, Cuzick J, Langsfeld E, Pearse A, Montoya GD, et al. A population-based study of human papillomavirus genotype prevalence in the United States: baseline measures prior to mass human papillomavirus vaccination. Int J Cancer. 2013;132(1):198-207. Epub 2012/04/26. doi: 10.1002/ijc.27608. PMID: 22532127; PMCID: PMC3852415.

22. Munoz N, Mendez F, Posso H, Molano M, van den Brule AJ, Ronderos M, et al. Incidence, duration, and determinants of cervical human papillomavirus infection in a cohort of Colombian women with normal cytological results. J Infect Dis. 2004;190(12):2077-87. PMID: 15551205.

23. Bratti MC, Rodriguez AC, Schiffman M, Hildesheim A, Morales J, Alfaro M, et al. Description of a seven-year prospective study of human papillomavirus infection and cervical neoplasia among 10000 women in Guanacaste, Costa Rica. Revista panamericana de salud publica = Pan American journal of public health. 2004;15(2):75-89. Epub 2004/03/20. PMID: 15030652.

24. Keefe KA, Schell MJ, Brewer C, McHale M, Brewster W, Chapman JA, et al. A randomized, double blind, Phase III trial using oral beta-carotene supplementation for women with high-grade cervical intraepithelial neoplasia. Cancer Epidemiol Biomarkers Prev. 2001;10(10):1029-35. PMID: 11588128.

25. Meyskens FL, Jr., Surwit E, Moon TE, Childers JM, Davis JR, Dorr RT, et al. Enhancement of regression of cervical intraepithelial neoplasia II (moderate dysplasia) with topically applied all-trans-retinoic acid: a randomized trial. J Natl Cancer Inst. 1994;86(7):539-43. PMID: 8133537.

26. Castle PE, Schiffman M, Wheeler CM, Solomon D. Evidence for frequent regression of cervical intraepithelial neoplasia-grade 2. Obstet Gynecol. 2009;113(1):18-25. doi: 10.1097/AOG.0b013e31818f5008. PMID: 19104355; PMCID: PMC2694845.

27. Moscicki AB, Ma Y, Wibbelsman C, Darragh TM, Powers A, Farhat S, et al. Rate of and risks for regression of cervical intraepithelial neoplasia 2 in adolescents and young women. Obstetrics and gynecology. 2010;116(6):1373-80. Epub 2010/11/26. doi: 10.1097/AOG.0b013e3181fe777f. PMID: 21099605; PMCID: PMC3057366.

28. Wang SM, Colombara D, Shi JF, Zhao FH, Li J, Chen F, et al. Six-year regression and progression of cervical lesions of different human papillomavirus viral loads in varied histological diagnoses. Int J Gynecol Cancer. 2013;23(4):716-23. doi: 10.1097/IGC.0b013e318286a95d. PMID: 23455757; PMCID: PMC3636161.

29. McCredie MR, Sharples KJ, Paul C, Baranyai J, Medley G, Jones RW, et al. Natural history of cervical neoplasia and risk of invasive cancer in women with cervical intraepithelial neoplasia 3: a retrospective cohort study. Lancet Oncol. 2008;9(5):425-34. Epub 2008/04/15. doi: 10.1016/S1470-2045(08)70103-7. PMID: 18407790.

30. McCrory D, Mather D, Bastian L, Datta S, Hasselblad V, Hickey J, et al. Evaluation of cervical cytology. Evidence report/technology assessment No. 5. AHCPR publication no. 99-E010 Rockville: Agency for Health Care Policy and Research; 1999.

31. National Cancer Institute. Surveillance, Epidemiology, End Results (SEER) Cancer Statistics Review, 1975-2013. <https://seer.cancer.gov/csr/1975_2013/> (last accessed August 1, 2019).

32. Hall MT, Simms KT, Lew JB, Smith MA, Brotherton JM, Saville M, et al. The projected timeframe until cervical cancer elimination in Australia: a modelling study. Lancet Public Health. 2019;4(1):e19-e27. Epub 2018/10/07. doi: 10.1016/S2468-2667(18)30183-X. PMID: 30291040.

33. Simms KT, Steinberg J, Caruana M, Smith MA, Lew JB, Soerjomataram I, et al. Impact of scaled up human papillomavirus vaccination and cervical screening and the potential for global elimination of cervical cancer in 181 countries, 2020-99: a modelling study. Lancet Oncol. 2019;20(3):394-407. Epub 2019/02/24. doi: 10.1016/S1470-2045(18)30836-2. PMID: 30795950.

34. Creighton P, Lew J, Clements M, Smith M, Howard K, Dyer S, et al. Cervical cancer screening in Australia: modelled evaluation of the impact of changing the recommended interval from two to three years. BMC Public Health. 2010;10:734.

35. Canfell K, Barnabas R, Patnick J, Beral V. The predicted effect of changes in cervical screening practice in the UK: results from a modelling study. Br J Cancer. 2004;91(3):530-6.

36. Canfell K, Clements M, Harris J. Cost-effectiveness of proposed changes to the national cervical screening program. 2008.

37. Canfell K, Lew JB, Smith M, Walker R. Cost-effectiveness modelling beyond MAVARIC study end-points. In: Kitchener HC, Blanks R, Cubie H, Desai M, Dunn G, Legood R, et al., editors. MAVARIC - a comparison of automation-assisted and manual cervical screening: a randomised controlled trial Health Technology Assessment 2011; Vol 15: No 32011.

38. Medical Services Advisory Committee. Automation Assisted and Liquid Based Cytology for Cervical Cancer Screening. MSAC reference 1122, Assessment report. Canberra: Australian Government Department of Health, 2009.

39. Canfell K, Shi JF, Lew JB, Walker R, Zhao FH, Simonella L, et al. Prevention of cervical cancer in rural China: Evaluation of HPV vaccination and primary HPV screening strategies. Vaccine. 2011;29(13):2487-94.

40. Shi JF, Canfell K, Lew JB, Zhao FH, Legood R, Ning Y, et al. Evaluation of primary HPV-DNA testing in relation to visual inspection methods for cervical cancer screening in rural China: an epidemiologic and cost-effectiveness modelling study. BMC Cancer. 2011;11(1):239.

41. Simms KT, Hanley SJB, Smith MA, Keane A, Canfell K. Impact of HPV vaccine hesitancy on cervical cancer in Japan: a modelling study. Lancet Public Health. 2020;5(4):e223-e34. Epub 2020/02/15. doi: 10.1016/S2468-2667(20)30010-4. PMID: 32057317.

42. Lew JB, Simms K, Smith MA, Kang YK, Xu XM, Caruana M, et al. National Cervical Screening Program Renewal: Effectiveness modelling and economic evaluation in the Australian setting. MSAC application number 1276 assessment report. Canberra: Department of Health, 2014.

43. Simms KT, Hall M, Smith MA, Lew J-B, Hughes S, Yuill S, et al. Optimal Management Strategies for Primary HPV Testing for Cervical Screening: Cost-Effectiveness Evaluation for the National Cervical Screening Program in Australia. PLoS One. 2017;12(1):e0163509. doi: 10.1371/journal.pone.0163509.

44. Lew J-B, Simms K, Smith M, Lewis H, Neal H, Canfell K. Effectiveness Modelling and Economic Evaluation of Primary HPV Screening for Cervical Cancer Prevention in New Zealand. PLoS One. 2016;11(5):e0151619. doi: 10.1371/journal.pone.0151619.

45. Kitchener HC, Canfell K, Gilham C, Sargent A, Roberts C, Desai M, et al. The clinical effectiveness and cost-effectiveness of primary human papillomavirus cervical screening in England: extended follow-up of the ARTISTIC randomised trial cohort through three screening rounds. Health Technol Assess. 2014;18(23):1-196.

46. Simms KT, Smith MA, Lew JB, Kitchener HC, Castle PE, Canfell K. Will cervical screening remain cost-effective in women offered the next generation nonavalent HPV vaccine? Results for four developed countries. Int J Cancer. 2016;139(12):2771-80.

47. Simms KT, Laprise J-F, Smith MA, Lew J-B, Caruana M, Brisson M, et al. Cost-effectiveness of the next generation nonavalent human papillomavirus vaccine in the context of primary human papillomavirus screening in Australia: a comparative modelling analysis. Lancet Public Health. 2017;1(2):e66-e75. doi: 10.1016/s2468-2667(16)30019-6.

48. Smith MA, Canfell K. Testing previous model predictions against new data on human papillomavirus vaccination program outcomes. BMC Res Notes. 2014;7(1):109. Epub 2014/02/27. doi: 10.1186/1756-0500-7-109. PMID: 24568634; PMCID: PMC3938033.

49. Canfell K, Simms K, Lew JB, Caruana M, Walker R, Smith M, et al., editors. Cost-effectiveness of primary HPV screening in England in unvaccinated and vaccinated cohorts: Evaluation based on ARTISTIC data. 28th International Human Papillomavirus Conference & Clinical and Public Health Workshops; 2012 December; San Juan, Puerto Rico.

50. Canfell K, Lew JB, Clements M, Smith M, Harris J, Simonella L, et al., editors. Impact of HPV vaccination on cost-effectiveness of existing screening programs: Example from New Zealand. 28th International Human Papillomavirus Conference & Clinical and Public Health Workshops; 2012 December; San Juan, Puerto Rico.

51. Hall MT, Simms KT, Lew J-B, Smith MA, Saville M, Canfell K. Projected future impact of HPV vaccination and primary HPV screening on cervical cancer rates from 2017–2035: Example from Australia. PLoS One. 2018;13(2):e0185332. doi: 10.1371/journal.pone.0185332. PMID: PMC5812553.

52. Smith MA, Gertig D, Hall M, Simms K, Lew J-B, Malloy M, et al. Transitioning from cytology-based screening to HPV-based screening at longer intervals: implications for resource use. BMC Health Serv Res. 2016;16(1):147.

53. Smith MA, Canfell K, Brotherton JM, Lew JB, Barnabas RV. The predicted impact of vaccination on human papillomavirus infections in Australia. Int J Cancer. 2008;123(8):1854-63.

54. Joste NE, Ronnett BM, Hunt WC, Pearse A, Langsfeld E, Leete T, et al. Human papillomavirus genotype-specific prevalence across the continuum of cervical neoplasia and cancer. Cancer Epidemiol Biomarkers Prev. 2015;24(1):230-40. doi: 10.1158/1055-9965.EPI-14-0775. PMID: 25363635; PMCID: PMC4294978.

55. Saraiya M, Unger ER, Thompson TD, Lynch CF, Hernandez BY, Lyu CW, et al. US assessment of HPV types in cancers: implications for current and 9-valent HPV vaccines. J Natl Cancer Inst. 2015;107(6):djv086. doi: 10.1093/jnci/djv086. PMID: 25925419; PMCID: PMC4838063.

56. Laskey PW, Meigs JW, Flannery JT. Uterine cervical carcinoma in Connecticut, 1935-1973: evidence for two classes of invasive disease. J Natl Cancer Inst. 1976;57(5):1037-43. PMID: 1003540.

57. Cuzick J, Myers O, Hunt WC, Robertson M, Joste NE, Castle PE, et al. A population-based evaluation of cervical screening in the United States: 2008-2011. Cancer Epidemiol Biomarkers Prev. 2014;23(5):765-73. Epub 2013/12/05. doi: 10.1158/1055-9965.EPI-13-0973. PMID: 24302677; PMCID: PMC4011954.

58. Cuzick J, Myers O, Hunt WC, Saslow D, Castle PE, Kinney W, et al. Human papillomavirus testing 2007-2012: Co-testing and triage utilization and impact on subsequent clinical management. Int J Cancer. 2015;136(12):2854-63. Epub 2014/12/03. doi: 10.1002/ijc.29337. PMID: 25447979.

59. Kinney W, Hunt WC, Dinkelspiel H, Robertson M, Cuzick J, Wheeler CM, et al. Cervical excisional treatment of young women: a population-based study. Gynecol Oncol. 2014;132(3):628-35. Epub 2014/01/08. doi: 10.1016/j.ygyno.2013.12.037. PMID: 24395062; PMCID: PMC3992337.

60. Chesson HW, Meites E, Ekwueme DU, Saraiya M, Markowitz LE. Cost-effectiveness of nonavalent HPV vaccination among males aged 22 through 26 years in the United States. Vaccine. 2018;36(29):4362-8. Epub 2018/06/12. doi: 10.1016/j.vaccine.2018.04.071. PMID: 29887325.

61. Chesson HW, Meites E, Ekwueme DU, Saraiya M, Markowitz LE. Cost-effectiveness of HPV vaccination for adults through age 45 years in the United States: Estimates from a simplified transmission model. Vaccine. 2020;38(50):8032-9. Epub 2020/10/31. doi: 10.1016/j.vaccine.2020.10.019. PMID: 33121846.

62. Burger EA, Kim JJ, Sy S, Castle PE. Age of Acquiring Causal Human Papillomavirus (HPV) Infections: Leveraging Simulation Models to Explore the Natural History of HPV-induced Cervical Cancer. Clin Infect Dis. 2017;65(6):893-9. Epub 2017/05/23. doi: 10.1093/cid/cix475. PMID: 28531261; PMCID: PMC5850533.

63. Brisson M, Bénard É, Drolet M, Bogaards JA, Baussano I, Vänskä S, et al. Population-level impact, herd immunity, and elimination after human papillomavirus vaccination: a systematic review and meta-analysis of predictions from transmission-dynamic models. Lancet Public Health. 2016;1(1). Epub 27/9/2016. doi: 10.1016/s2468-2667(16)30001-9.

64. Garland SM, Steben M, Sings HL, James M, Lu S, Railkar R, et al. Natural history of genital warts: analysis of the placebo arm of 2 randomized phase III trials of a quadrivalent human papillomavirus (types 6, 11, 16, and 18) vaccine. J Infect Dis. 2009;199(6):805-14.

65. Neumann PJ, Cohen JT, Weinstein MC. Updating cost-effectiveness--the curious resilience of the $50,000-per-QALY threshold. N Engl J Med. 2014;371(9):796-7. Epub 2014/08/28. doi: 10.1056/NEJMp1405158. PMID: 25162885.

66. Centers for Disease Control and Prevention. National, State and Local Area Vaccination Coverage among Adolescents Aged 13 - 17 Years - United States 2008. MMWR Morb Mortal Wkly Rep. 2009;58(36):997-1001.

67. Centers for Disease Control and Prevention. National, State and Local Area Vaccination Coverage among Adolescents Aged 13 - 17 Years - United States 2009. MMWR Morb Mortal Wkly Rep. 2010;59(32):1018-23.

68. Centers for Disease Control and Prevention. National, State and Local Area Vaccination Coverage among Adolescents Aged 13 through 17 Years - United States 2010. MMWR Morb Mortal Wkly Rep. 2011;60(33):1117-23.

69. Centers for Disease Control and Prevention. National and state vaccination coverage among adolescents aged 13-17 years--United States, 2012. MMWR Morb Mortal Wkly Rep. 2013;62(34):685-93. Epub 2013/08/30. PMID: 23985496.

70. Reagan-Steiner S, Yankey D, Jeyarajah J, Elam-Evans LD, Singleton JA, Curtis CR, et al. National, Regional, State, and Selected Local Area Vaccination Coverage Among Adolescents Aged 13-17 Years--United States, 2014. MMWR Morb Mortal Wkly Rep. 2015;64(29):784-92. PMID: 26225476.

71. Reagan-Steiner S, Yankey D, Jeyarajah J, Elam-Evans LD, Curtis CR, MacNeil J, et al. National, Regional, State, and Selected Local Area Vaccination Coverage Among Adolescents Aged 13-17 Years - United States, 2015. MMWR Morb Mortal Wkly Rep. 2016;65(33):850-8. doi: 10.15585/mmwr.mm6533a4. PMID: 27561081.

72. Petrosky E, Bocchini JA, Jr., Hariri S, Chesson H, Curtis CR, Saraiya M, et al. Use of 9-valent human papillomavirus (HPV) vaccine: updated HPV vaccination recommendations of the advisory committee on immunization practices. MMWR Morb Mortal Wkly Rep. 2015;64(11):300-4. Epub 2015/03/27. PMID: 25811679; PMCID: PMC4584883.

73. Saslow D, Solomon D, Lawson HW, Killackey M, Kulasingam SL, Cain J, et al. American Cancer Society, American Society for Colposcopy and Cervical Pathology, and American Society for Clinical Pathology screening guidelines for the prevention and early detection of cervical cancer. CA Cancer J Clin. 2012;62(3):147-72. Epub 2012/03/17. doi: 10.3322/caac.21139. PMID: 22422631; PMCID: PMC3801360.

74. Curry SJ, Krist AH, Owens DK, Barry MJ, Caughey AB, Davidson KW, et al. Screening for Cervical Cancer: US Preventive Services Task Force Recommendation Statement. JAMA. 2018;320(7):674-86. Epub 2018/08/25. doi: 10.1001/jama.2018.10897. PMID: 30140884.

75. Massad LS, Einstein MH, Huh WK, Katki HA, Kinney WK, Schiffman M, et al. 2012 updated consensus guidelines for the management of abnormal cervical cancer screening tests and cancer precursors. J Low Genit Tract Dis. 2013;17(5 Suppl 1):S1-S27. Epub 2013/03/27. doi: 10.1097/LGT.0b013e318287d329. PMID: 23519301.

76. Drolet M, Brisson M, Maunsell E, Franco EL, Coutlee F, Ferenczy A, et al. The psychosocial impact of an abnormal cervical smear result. Psychooncology. 2012;21(10):1071-81.

77. Kim JJ, Wright TC, Goldie SJ. Cost-effectiveness of alternative triage strategies for atypical squamous cells of undetermined significance. JAMA. 2002;287(18):2382-90.

78. Gold MR, Franks P, McCoy KI, Fryback DG. Toward consistency in cost-utility analyses: using national measures to create condition-specific values. Med Care. 1998;36(6):778-92. PMID: 9630120.

**Table A.** Comparison of main model attributes

|  | **Harvard** | **Policy1-Cervix** |
| --- | --- | --- |
| **Model Attributes** | | |
| Dynamic (interactive) or static (non-interactive) | Dynamic (HPV transmission/ vaccination)  Static (cervical cancer natural history, screening, follow-up) | Dynamic (HPV transmission/ vaccination)  Static (cervical cancer natural history, screening, follow-up) |
| Mode of analysis, simulating life histories | Individual-based | Individual-based |
| Cycle length | Monthly | 6 or 12-monthly |
| **HPV Transmission and Infection** | | |
| HPV types included | HPV16, HPV18, HPV31, HPV33, HPV45, HPV52, HPV58, pooled other high-risk, pooled low-risk | HPV16, HPV18,  pooled HPV31/33/45/52/58, pooled other high-risk (non-9v) |
| Natural Immunity | Lifelong, reduced probability of future type-specific infection | Waning reduced probability of future type-specific infection |
| **Cervical Carcinogenesis** | | |
| Health states included | Healthy, HPV, CIN2, CIN3, Cancer (stage-specific; SCC only) | Healthy, HPV, CIN1, CIN2, CIN3, Cancer (stage-specific; all cancers) |
| Progression and regression rates | Age-specific, time since HPV infection or lesion development | Age-specific |
| **Model Calibration** | | |
| Calibrated parameters | HPV incidence, HPV progression, CIN progression and regression rates; HPV natural immunity; cancer symptom detection; progression of undetected asymptomatic cancer by stage | HPV and CIN progression and regression rates; HPV natural immunity; cancer symptom detection; progression of undetected asymptomatic cancer by stage |
| **Cancer control Interventions (adjustable parameters)** | | |
| Vaccination | Yes | Yes |
| Screening | Yes | Yes |
| Diagnosis | Yes | Yes |
| Treatment | Yes | Yes |
| Abbreviations: CIN, cervical intraepithelial neoplasia; HPV, human papillomavirus; SCC, squamous cell carcinoma. | | |

**Table B.** Annual number of total partnerships by age and sexual activity category, males [18]

|  | **Sexual Activity Category** | | | |
| --- | --- | --- | --- | --- |
| **Age (years)** | **1** | **2** | **3** | **4** |
| 0-11 | 0 | 0 | 0 | 0 |
| 12-14 | 0 | 0 | 1 | 3 |
| 15-19 | 0 | 1 | 3 | 9 |
| 20-39 | 0 | 1 | 3 | 11 |
| 40-44 | 0 | 1 | 3 | 12 |
| 45-59 | 0 | 1 | 3 | 13 |
| 60+ | 0 | 0 | 0 | 0 |

**Table C.** Annual number of total partnerships by age and sexual activity category, females [18]

|  | **Sexual Activity Category** | | | |
| --- | --- | --- | --- | --- |
| **Age (years)** | **1** | **2** | **3** | **4** |
| 0-11 | 0 | 0 | 0 | 0 |
| 12-14 | 0 | 0 | 1 | 3 |
| 15-19 | 0 | 1 | 3 | 8 |
| 20-59 | 0 | 1 | 3 | 10 |
| 60+ | 0 | 0 | 0 | 0 |

**Table D.** Average partnership duration in months (95% CI) by age and sexual activity category [18]

|  | **Sexual Activity Category** | | | |
| --- | --- | --- | --- | --- |
| **Age (years)** | **1** | **2** | **3** | **4** |
| 0-11 | 0 | 0 | 0 | 0 |
| 12-14 | 0 | 0 | 9.5 (7.7, 11.3) | 8 (5.1, 11.0) |
| 15-19 | 0 | 12.6 (10.3, 14.9) | 9.5 (7.7, 11.3) | 8 (5.1, 11.0) |
| 20-24 | 0 | 31.1 (28.1, 34.1) | 19.3 (15.1, 23.5) | 10.1 (6.5, 13.7) |
| 25-29 | 0 | 54.9 (51.6, 58.1) | 27.6 (20.3, 34.8) | 27.4 (15.7, 39.1) |
| 30-34 | 0 | 91.1 (86.7, 95.4) | 30.9 (20.9, 40.9) | 32.4 (12.2, 52.6) |
| 35-39 | 0 | 118.9 (113.1, 124.7) | 41.4 (27.8, 55.0) | 19.3 (12.1, 26.5) |
| 40+ | 0 | 154.9 (146.1, 163.6) | 75.9 (47.0, 104.9) | 24.9 (5.3, 44.6) |

**Table E.** Assortativeness [18]

| **By age** | **Proportion** |
| --- | --- |
| Same age category | 0.55 |
| One age category younger | 0.30 |
| One age category older | 0.11 |
| Other ages | 0.04 |
| **By sexual activity category** | **Proportion** |
| Same SAC | 0.55 |
| Other SAC | 0.45 |

**Table F.** HPV clearance by time since infection [19,20]

| **Duration (months)** | **HPV 16** | **HPV 18** | **HPV 31** | **HPV 33** | **HPV 45** | **HPV 52** | **HPV 58** |
| --- | --- | --- | --- | --- | --- | --- | --- |
| Females | | | | | | | |
| 1 | 0.04189 | 0.07334 | 0.06345 | 0.08345 | 0.07852 | 0.06300 | 0.06557 |
| 16 | 0.04075 | 0.06324 | 0.03383 | 0.04496 | 0.04258 | 0.04440 | 0.05443 |
| 28 | 0.03391 | 0.05361 | 0.03383 | 0.03616 | 0.04168 | 0.04440 | 0.05397 |
| 40 | 0.03189 | 0.02062 | 0.03383 | 0.03616 | 0.03013 | 0.03933 | 0.03332 |
| 50 | 0.03189 | 0.02062 | 0.03383 | 0.03616 | 0.01507 | 0.03933 | 0.01666 |
| 52+ | 0.01985 | 0.02062 | 0.03383 | 0.03616 | 0.01507 | 0.03933 | 0.01666 |
| Males | | | | | | | |
| 1 | 0.06283 | 0.11001 | 0.09517 | 0.12518 | 0.11778 | 0.09450 | 0.09836 |
| 16 | 0.06113 | 0.09485 | 0.05074 | 0.06743 | 0.06387 | 0.06660 | 0.08165 |
| 28 | 0.05086 | 0.08041 | 0.05074 | 0.05423 | 0.06251 | 0.06660 | 0.08095 |
| 40 | 0.04783 | 0.03092 | 0.05074 | 0.05423 | 0.04520 | 0.05899 | 0.04998 |
| 50 | 0.04783 | 0.03092 | 0.05074 | 0.05423 | 0.02261 | 0.05899 | 0.02499 |
| 52+ | 0.02977 | 0.03092 | 0.05074 | 0.05423 | 0.02261 | 0.05899 | 0.02499 |

**Table G.** Harvard calibrated parameter set for dynamic model

| **Calibration parameter** | **Search range** | **Best-Fitting Parameter Sets** |
| --- | --- | --- |
| **Female to male HPV transmission, monthly per partner** | | |
| HPV 16 | 0.01-0.50 | 0.10628 |
| HPV 18 | 0.01-0.50 | 0.22027 |
| HPV 31 | 0.01-0.50 | 0.08058 |
| HPV 33 | 0.01-0.50 | 0.15168 |
| HPV 45 | 0.01-0.50 | 0.08079 |
| HPV 52 | 0.01-0.50 | 0.08267 |
| HPV 58 | 0.01-0.50 | 0.07950 |
| **Male to female HPV transmission, monthly per partner** | | |
| HPV 16 | 0.01-female | 0.09978 |
| HPV 18 | 0.01-female | 0.06117 |
| HPV 31 | 0.01-female | 0.06649 |
| HPV 33 | 0.01-female | 0.02782 |
| HPV 45 | 0.01-female | 0.07683 |
| HPV 52 | 0.01-female | 0.08067 |
| HPV 58 | 0.01-female | 0.06817 |
| **Natural immunity, females** | | |
| HPV 16 | 0.10-0.50 | 0.47953 |
| HPV 18 | 0.10-0.50 | 0.46498 |
| HPV 31 | 0.10-0.50 | 0.45256 |
| HPV 33 | 0.10-0.50 | 0.49500 |
| HPV 45 | 0.10-0.50 | 0.48493 |
| HPV 52 | 0.10-0.50 | 0.35680 |
| HPV 58 | 0.10-0.50 | 0.49428 |
| **Natural immunity, males** | | |
| HPV 16 | 0.00-0.10 | 0.03072 |
| HPV 18 | 0.00-0.10 | 0.08347 |
| HPV 31 | 0.00-0.10 | 0.09986 |
| HPV 33 | 0.00-0.10 | 0.01589 |
| HPV 45 | 0.00-0.10 | 0.07902 |
| HPV 52 | 0.00-0.10 | 0.05840 |
| HPV 58 | 0.00-0.10 | 0.04074 |

**Table H.** Harvard calibrated parameter set for stochastic model*****

| **Model parameter** | **Best-fitting parameter value** | |
| --- | --- | --- |
| **HPV 16 incidence** [19,22] | | |
| Age <20 years | 0.00003-0.00478 | |
| Age 20-24 years | 0.00277-0.00384 | |
| Age 25-29 years | 0.00240-0.00267 | |
| Age 30-34 years | 0.00223-0.00240 | |
| Age 35-39 years | 0.00216-0.00221 | |
| Age 40-44 years | 0.00203-0.00210 | |
| Age 45-49 years | 0.00185-0.00201 | |
| Age 50-54 years | 0.00167-0.00181 | |
| Age 55-59 years | 0.00151-0.00164 | |
| Age 60+ years | 0.00067-0.00148 | |
| **HPV 18 incidence** [19,22] | | |
| Age <20 years | 0.00002-0.00417 | |
| Age 20-24 years | 0.00161-0.00172 | |
| Age 25-29 years | 0.00088-0.00146 | |
| Age 30-34 years | 0.00073-0.00085 | |
| Age 35-39 years | 0.00057-0.00070 | |
| Age 40-44 years | 0.00048-0.00056 | |
| Age 45-49 years | 0.00044-0.00048 | |
| Age 50-54 years | 0.00040-0.00043 | |
| Age 55-59 years | 0.00036-0.00039 | |
| Age 60+ years | 0.00016-0.00035 | |
| **HPV 31 incidence** [19,22] | | |
| Age <20 years | 0.00001-0.00249 | |
| Age 20-24 years | 0.00157-0.00238 | |
| Age 25-29 years | 0.00105-0.00140 | |
| Age 30-34 years | 0.00073-0.00096 | |
| Age 35-39 years | 0.00059-0.00066 | |
| Age 40-44 years | 0.00057-0.00058 | |
| Age 45-49 years | 0.00053-0.00057 | |
| Age 50-54 years | 0.00048-0.00051 | |
| Age 55-59 years | 0.00043-0.00047 | |
| Age 60+ years | 0.00019-0.00042 | |
| **HPV 33 incidence** [19,22] | | |
| Age <20 years | 0.00001-0.00149 | |
| Age 20-24 years | 0.00078-0.00131 | |
| Age 25-29 years | 0.00048-0.00068 | |
| Age 30-34 years | 0.00035-0.00042 | |
| Age 35-39 years | 0.00030-0.00033 | |
| Age 40-44 years | 0.00028-0.00030 | |
| Age 45-49 years | 0.00025-0.00027 | |
| Age 50-54 years | 0.00022-0.00024 | |
| Age 55-59 years | 0.00021-0.00022 | |
| Age 60+ years | 0.00010-0.00021 | |
| **HPV 45 incidence** [19,22] | | |
| Age <20 years | 0.00001-0.00137 | |
| Age 20-24 years | 0.00056-0.00109 | |
| Age 25-29 years | 0.00043-0.00051 | |
| Age 30-34 years | 0.00037-0.00042 | |
| Age 35-39 years | 0.00030-0.00036 | |
| Age 40-44 years | 0.00025-0.00029 | |
| Age 45-49 years | 0.00022-0.00024 | |
| Age 50-54 years | 0.00019-0.00021 | |
| Age 55-59 years | 0.00017-0.00018 | |
| Age 60+ years | 0.00007-0.00017 | |
| **HPV 52 incidence** [19,22] | | |
| Age <20 years | 0.00002-0.00461 | |
| Age 20-24 years | 0.00105-0.00311 | |
| Age 25-29 years | 0.00071-0.00099 | |
| Age 30-34 years | 0.00065-0.00067 | |
| Age 35-39 years | 0.00061-0.00064 | |
| Age 40-44 years | 0.00058-0.00061 | |
| Age 45-49 years | 0.00059-0.00063 | |
| Age 50-54 years | 0.00053-0.00057 | |
| Age 55-59 years | 0.00048-0.00052 | |
| Age 60+ years | 0.00021-0.00047 | |
| **HPV 58 incidence** [19,22] | | |
| Age <20 years | 0.00001-0.00184 | |
| Age 20-24 years | 0.00091-0.00170 | |
| Age 25-29 years | 0.00072-0.00085 | |
| Age 30-34 years | 0.00062-0.00070 | |
| Age 35-39 years | 0.00053-0.00060 | |
| Age 40-44 years | 0.00042-0.00050 | |
| Age 45-49 years | 0.00038-0.00041 | |
| Age 50-54 years | 0.00034-0.00037 | |
| Age 55-59 years | 0.00031-0.00033 | |
| Age 60+ years | 0.00008-0.00030 | |
| **Other carcinogenic incidence** [19,22] | | |
| Age <20 years | 0.00004-0.01863 | |
| Age 20-24 years | 0.01361-0.01735 | |
| Age 25-29 years | 0.00905-0.01282 | |
| Age 30-34 years | 0.00664-0.00830 | |
| Age 35-39 years | 0.00558-0.00618 | |
| Age 40-44 years | 0.00490-0.00543 | |
| Age 45-49 years | 0.00454-0.00486 | |
| Age 50-54 years | 0.00411-0.00445 | |
| Age 55-59 years | 0.00372-0.00403 | |
| Age 60+ years | 0.00164-0.00364 | |
| **Non-carcinogenic incidence** [19,22] | | |
| Age <20 years | 0.00006-0.03109 | |
| Age 20-24 years | 0.01105-0.01135 | |
| Age 25-29 years | 0.00866-0.01083 | |
| Age 30-34 years | 0.00221-0.00266 | |
| Age 35-39 years | 0.00201-0.00216 | |
| Age 40-44 years | 0.00174-0.00196 | |
| Age 45-49 years | 0.00121-0.00161 | |
| Age 50-54 years | 0.00091-0.00114 | |
| Age 55-59 years | 0.00066-0.00086 | |
| Age 60+ years | 0.00031-0.00064 | |
| **Natural immunity** ^†^ | 0.73222 | |
| **HPV 16 clearance** ^‡^ [22,23] | | |
| Year 1 | 0.04189 | |
| Year 2 | 0.04075 | |
| Year 3 | 0.03390 | |
| Year 4 | 0.03189 | |
| Year 5+ | 0.01985 | |
| **HPV 18 clearance** ^‡^ [22,23] | | |
| Year 1 | 0.07334 | |
| Year 2 | 0.06324 | |
| Year 3 | 0.05360 | |
| Year 4 | 0.02062 | |
| Year 5+ | 0.02062 | |
| **HPV 31 clearance** ^‡^ [22,23] | | |
| Year 1 | 0.06345 | |
| Year 2 | 0.03383 | |
| Year 3 | 0.03383 | |
| Year 4 | 0.03383 | |
| Year 5+ | 0.03383 | |
| **HPV 33 clearance** ^‡^ [22,23] | | |
| Year 1 | 0.08345 | |
| Year 2 | 0.04496 | |
| Year 3 | 0.03616 | |
| Year 4 | 0.03616 | |
| Year 5+ | 0.03616 | |
| **HPV 45 clearance** ^‡^ [22,23] | | |
| Year 1 | 0.07852 | |
| Year 2 | 0.04258 | |
| Year 3 | 0.04168 | |
| Year 4 | 0.03013 | |
| Year 5+ | 0.01507 | |
| **HPV 52 clearance** ^‡^ [22,23] | | |
| Year 1 | 0.06300 | |
| Year 2 | 0.04440 | |
| Year 3 | 0.04440 | |
| Year 4 | 0.03933 | |
| Year 5+ | 0.03933 | |
| **HPV 58 clearance** ^‡^ [22,23] | | |
| Year 1 | 0.06557 | |
| Year 2 | 0.05443 | |
| Year 3 | 0.05397 | |
| Year 4 | 0.03332 | |
| Year 5+ | 0.01666 | |
| **Other carcinogenic clearance** ^‡^ [22,23] | | |
| Year 1 | 0.08077 | |
| Year 2 | 0.06663 | |
| Year 3 | 0.05397 | |
| Year 4 | 0.04923 | |
| Year 5+ | 0.00509 | |
| **Non-carcinogenic clearance** ^‡^ [22-24] | | |
| Year 1 | 0.05189 | |
| Year 2 | 0.05001 | |
| Year 3 | 0.03465 | |
| Year 4 | 0.03465 | |
| Year 5+ | 0.02861 | |
| **HPV 16 progression to CIN2** ^‡^ [22,23] | | |
| Year 1 | 0.00171 | |
| Year 2 | 0.00242 | |
| Year 3 | 0.00258 | |
| Year 4 | 0.00552 | |
| Year 5 | 0.01500 | |
| Years 6-10 | 0.02941 | |
| Years 11+ | 0.04835 | |
| **HPV 16 progression to CIN3** ^‡^ [22,23] | | |
| Year 1 | 0.00057 | |
| Year 2 | 0.00081 | |
| Year 3 | 0.00086 | |
| Year 4 | 0.00184 | |
| Year 5 | 0.00502 | |
| Years 6-10 | 0.00985 | |
| Years 11+ | 0.01620 | |
| **HPV 18 progression to CIN2** ^‡^ [22,23] | | |
| Year 1 | 0.00004 | |
| Year 2 | 0.00019 | |
| Year 3 | 0.00019 | |
| Year 4 | 0.00773 | |
| Year 5 | 0.00773 | |
| Years 6-10 | 0.01516 | |
| Years 11+ | 0.02493 | |
| **HPV 18 progression to CIN3** ^‡^ [22,23] | | |
| Year 1 | 0.00001 | |
| Year 2 | 0.00005 | |
| Year 3 | 0.00005 | |
| Year 4 | 0.00194 | |
| Year 5 | 0.00194 | |
| Years 6-10 | 0.00380 | |
| Years 11+ | 0.00625 | |
| **HPV 31 progression to CIN2** ^‡^ [22,23] | | |
| Year 1 | 0.00026 | |
| Year 2 | 0.00278 | |
| Year 3 | 0.00309 | |
| Year 4 | 0.00693 | |
| Year 5 | 0.00693 | |
| Years 6-10 | 0.01359 | |
| Years 11+ | 0.02234 | |
| **HPV 31 progression to CIN3** ^‡^ [22,23] | | |
| Year 1 | 0.00007 | |
| Year 2 | 0.00070 | |
| Year 3 | 0.00077 | |
| Year 4 | 0.00174 | |
| Year 5 | 0.00174 | |
| Years 6-10 | 0.00341 | |
| Years 11+ | 0.00560 | |
| **HPV 33 progression to CIN2** ^‡^ [22,23] | | |
| Year 1 | 0.00072 | |
| Year 2 | 0.00072 | |
| Year 3 | 0.00494 | |
| Year 4 | 0.00494 | |
| Year 5 | 0.00494 | |
| Years 6-10 | 0.00968 | |
| Years 11+ | 0.01592 | |
| **HPV 33 progression to CIN3** ^‡^ [22,23] | | |
| Year 1 | 0.00018 | |
| Year 2 | 0.00018 | |
| Year 3 | 0.00124 | |
| Year 4 | 0.00124 | |
| Year 5 | 0.00124 | |
| Years 6-10 | 0.00243 | |
| Years 11+ | 0.00399 | |
| **HPV 45 progression to CIN2** ^‡^ [22,23] | | |
| Year 1 | 0.00000 | |
| Year 2 | 0.00000 | |
| Year 3 | 0.00226 | |
| Year 4 | 0.00533 | |
| Year 5 | 0.00533 | |
| Years 6-10 | 0.01046 | |
| Years 11+ | 0.01719 | |
| **HPV 45 progression to CIN3** ^‡^ [22,23] | | |
| Year 1 | 0.00000 | |
| Year 2 | 0.00000 | |
| Year 3 | 0.00056 | |
| Year 4 | 0.00134 | |
| Year 5 | 0.00134 | |
| Years 6-10 | 0.00262 | |
| Years 11+ | 0.00431 | |
| **HPV 52 progression to CIN2** ^‡^ [22,23] | | |
| Year 1 | 0.00088 | |
| Year 2 | 0.00168 | |
| Year 3 | 0.00168 | |
| Year 4 | 0.00198 | |
| Year 5 | 0.00568 | |
| Years 6-10 | 0.01113 | |
| Years 11+ | 0.01831 | |
| **HPV 52 progression to CIN3** ^‡^ [22,23] | | |
| Year 1 | 0.00022 | |
| Year 2 | 0.00042 | |
| Year 3 | 0.00042 | |
| Year 4 | 0.00049 | |
| Year 5 | 0.00142 | |
| Years 6-10 | 0.00279 | |
| Years 11+ | 0.00459 | |
| **HPV 58 progression to CIN2** ^‡^ [22,23] | | |
| Year 1 | 0.00059 | |
| Year 2 | 0.00247 | |
| Year 3 | 0.00247 | |
| Year 4 | 0.00462 | |
| Year 5 | 0.01025 | |
| Years 6-10 | 0.02009 | |
| Years 11+ | 0.03303 | |
| **HPV 58 progression to CIN3** ^‡^ [22,23] | | |
| Year 1 | 0.00015 | |
| Year 2 | 0.00062 | |
| Year 3 | 0.00062 | |
| Year 4 | 0.00116 | |
| Year 5 | 0.00257 | |
| Years 6-10 | 0.00504 | |
| Years 11+ | 0.00829 | |
| **Other carcinogenic HPV progression to CIN2** ^‡^ [22,23] | | |
| Year 1 | | 0.00013 |
| Year 2 | | 0.00037 |
| Year 3 | | 0.00196 |
| Year 4 | | 0.00196 |
| Year 5 | | 0.00196 |
| Years 6-10 | | 0.00384 |
| Years 11+ | | 0.00632 |
| **Other carcinogenic HPV progression to CIN3** ^‡^ [22,23] | | |
| Year 1 | | 0.00003 |
| Year 2 | | 0.00009 |
| Year 3 | | 0.00049 |
| Year 4 | | 0.00049 |
| Year 5 | | 0.00049 |
| Years 6-10 | | 0.00096 |
| Years 11+ | | 0.00158 |
| **Non-carcinogenic HPV progression to CIN2** ^‡^ [22,23] | | |
| Year 1 | | 0.00021 |
| Year 2 | | 0.00029 |
| Year 3 | | 0.00031 |
| Year 4 | | 0.00066 |
| Year 5 | | 0.00066 |
| Years 6-10 | | 0.00130 |
| Years 11+ | | 0.00214 |
| **Non-carcinogenic HPV progression to CIN3** ^‡^ [22,23] | | |
| Year 1 | | 0.00002 |
| Year 2 | | 0.00003 |
| Year 3 | | 0.00003 |
| Year 4 | | 0.00007 |
| Year 5 | | 0.00007 |
| Years 6-10 | | 0.00014 |
| Years 11+ | | 0.00024 |
| **Regression of CIN2 related to HPV 16** ^§^ [22,25-29] | | |
| Years 1-5 | | 0.04500 |
| Years 6-10 | | 0.03600 |
| Years 11-20 | | 0.02700 |
| Years 21-30 | | 0.00180 |
| Years 31-40 | | 0.00090 |
| Years 41+ | | 0.00045 |
| **Regression of CIN2 related to other HR HPV** ^§^ [22,25-29] | | |
| Years 1-5 | | 0.05000 |
| Years 6-10 | | 0.04000 |
| Years 11-20 | | 0.03000 |
| Years 21-30 | | 0.00200 |
| Years 31-40 | | 0.00100 |
| Years 41+ | | 0.00050 |
| **Regression of CIN3 related to HPV 16** ^§^ [22,25-29] | | |
| Years 1-5 | | 0.02250 |
| Years 6-10 | | 0.01800 |
| Years 11-20 | | 0.01350 |
| Years 21-30 | | 0.00090 |
| Years 31-40 | | 0.00045 |
| Years 41+ | | 0.00023 |
| **Regression of CIN3 related to other HR HPV** ^§^ [22,25-29] | | |
| Years 1-5 | | 0.02500 |
| Years 6-10 | | 0.02000 |
| Years 11-20 | | 0.01500 |
| Years 21-30 | | 0.00100 |
| Years 31-40 | | 0.00050 |
| Years 41+ | | 0.00025 |
| **Progression of CIN2 to invasive cancer related to HPV 16** ^¶^ [22,30] | | |
| Years 1-5 | | 0.00004 |
| Years 6-10 | | 0.00004 |
| Years 11-20 | | 0.00100 |
| Years 21-29 | | 0.00293 |
| Years 30-34 | | 0.00585 |
| Years 35-39 | | 0.00634 |
| Years 40-44 | | 0.01365 |
| Years 45-49 | | 0.01463 |
| Years 50+ | | 0.08756 |
| **Progression of CIN2 to invasive cancer related to HPV 18** ^¶^ [22,30] | | |
| Years 1-5 | | 0.00004 |
| Years 6-10 | | 0.00004 |
| Years 11-20 | | 0.00107 |
| Years 21-29 | | 0.00313 |
| Years 30-34 | | 0.00625 |
| Years 35-39 | | 0.00677 |
| Years 40-44 | | 0.01459 |
| Years 45-49 | | 0.01563 |
| Years 50+ | | 0.09357 |
| **Progression of CIN2 to invasive cancer related to HPV 33** ^¶^ [22,30] | | |
| Years 1-5 | | 0.00004 |
| Years 6-10 | | 0.00004 |
| Years 11-20 | | 0.00103 |
| Years 21-29 | | 0.00300 |
| Years 30-34 | | 0.00601 |
| Years 35-39 | | 0.00651 |
| Years 40-44 | | 0.01402 |
| Years 45-49 | | 0.01502 |
| Years 50+ | | 0.08995 |
| **Progression of CIN2 to invasive cancer related to HPV 31/45/52/58** ^¶^ [22,30] | | |
| Years 1-5 | | 0.00002 |
| Years 6-10 | | 0.00002 |
| Years 11-20 | | 0.00057 |
| Years 21-29 | | 0.00167 |
| Years 30-34 | | 0.00334 |
| Years 35-39 | | 0.00362 |
| Years 40-44 | | 0.00780 |
| Years 45-49 | | 0.00835 |
| Years 50+ | | 0.05000 |
| **Progression of CIN2 to invasive cancer related to other HR HPV** ^¶^ [22,30] | | |
| Years 1-5 | | 0.00000 |
| Years 6-10 | | 0.00000 |
| Years 11-20 | | 0.00011 |
| Years 21-29 | | 0.00033 |
| Years 30-34 | | 0.00066 |
| Years 35-39 | | 0.00071 |
| Years 40-44 | | 0.00153 |
| Years 45-49 | | 0.00164 |
| Years 50+ | | 0.00164 |
| **Progression of CIN3 to invasive cancer related to HPV 16** ^¶^ [22,30] | | |
| Years 1-5 | | 0.00019 |
| Years 6-10 | | 0.00020 |
| Years 11-20 | | 0.00483 |
| Years 21-29 | | 0.01412 |
| Years 30-34 | | 0.02823 |
| Years 35-39 | | 0.03058 |
| Years 40-44 | | 0.06587 |
| Years 45-49 | | 0.07058 |
| Years 50+ | | 0.08450 |
| **Progression of CIN3 to invasive cancer related to HPV 18** ^¶^ [22,30] | | |
| Years 1-5 | | 0.00021 |
| Years 6-10 | | 0.00023 |
| Years 11-20 | | 0.00545 |
| Years 21-29 | | 0.01595 |
| Years 30-34 | | 0.03189 |
| Years 35-39 | | 0.03455 |
| Years 40-44 | | 0.07442 |
| Years 45-49 | | 0.07973 |
| Years 50+ | | 0.09547 |
| **Progression of CIN3 to invasive cancer related to HPV 33** ^¶^ [22,30] | | |
| Years 1-5 | | 0.00017 |
| Years 6-10 | | 0.00018 |
| Years 11-20 | | 0.00432 |
| Years 21-29 | | 0.01262 |
| Years 30-34 | | 0.02525 |
| Years 35-39 | | 0.02735 |
| Years 40-44 | | 0.05891 |
| Years 45-49 | | 0.06312 |
| Years 50+ | | 0.07558 |
| **Progression of CIN3 to invasive cancer related to HPV 31/45/52/58** ^¶^ [22,30] | | |
| Years 1-5 | | 0.00011 |
| Years 6-10 | | 0.00012 |
| Years 11-20 | | 0.00286 |
| Years 21-29 | | 0.00835 |
| Years 30-34 | | 0.01670 |
| Years 35-39 | | 0.01810 |
| Years 40-44 | | 0.03898 |
| Years 45-49 | | 0.04176 |
| Years 50+ | | 0.05000 |
| **Progression of CIN3 to invasive cancer related to other HR HPV** ^¶^ [22,30] | | |
| Years 1-5 | | 0.00001 |
| Years 6-10 | | 0.00001 |
| Years 11-20 | | 0.00031 |
| Years 21-29 | | 0.00091 |
| Years 30-34 | | 0.00182 |
| Years 35-39 | | 0.00197 |
| Years 40-44 | | 0.00424 |
| Years 45-49 | | 0.00454 |
| Years 50+ | | 0.00454 |
| **Progression of invasive cancer stages** [30,31] | | |
| Local to regional | | 0.02000 |
| Regional to distant | | 0.02500 |
| **Invasive cancer mortality** ǁ [31] | | |
| Local | |  |
| Year 1 | | 0.00159 |
| Years 2-3 | | 0.00141 |
| Years 4-20 | | 0.00094 |
| Regional | |  |
| Year 1 | | 0.00946 |
| Years 2-3 | | 0.00781 |
| Years 4-20 | | 0.00362 |
| Distant | |  |
| Year 1 | | 0.02934 |
| Years 2-3 | | 0.01947 |
| Years 4-20 | | 0.00760 |
| **Probability of symptom detection** [30,31] | | |
| Local | | 0.01740 |
| Regional | | 0.07350 |
| Distant | | 0.17460 |
| Abbreviations: CIN2, cervical intraepithelial neoplasia grade 2; CIN3, CIN grade 3; HPV, human papillomavirus; HR, high-risk. | | |
| * Values represent monthly probabilities, unless otherwise noted; range represents minimum and maximum values within the age group. | | |
| ^†^ Natural immunity represents the percentage reduction in risk of subsequent, type-specific infection after a woman has cleared a carcinogenic infection with the same type. Risk reduction is assumed to be lifelong and constant across age, time, and genotype. | | |
| ^‡^ HPV clearance and progression probabilities are a function of time since infection (i.e., persistence). | | |
| ^§^ Precancer regression probabilities decrease by time since lesion onset and are constant across carcinogenic HPV types. Given limited data, we assumed that the monthly CIN3 regression probability is 50% of CIN2 regression; 50% regress to type-specific HPV-infected health states and 50% regress to the Normal health state. | | |
| ^¶^ Precancer progression probabilities increase by time since lesion onset and are constant across carcinogenic HPV types. CIN2 progression is set at 20% of CIN3 progression (for carcinogenic types only). | | |
| ǁ In addition to time since diagnosis, cancer mortality was adjusted for age at diagnosis by applying stage-specific multipliers to the baseline probabilities that ranged from 0.30 to 7.39 for local cancer; 0.39 to 1.30 for regional cancer; and 0.002 to 15.16 for distant cancer [31]. | | |

**Table I.** Annual vulvar cancer incidence rates (per 100,000) [60,61]

| Age (years) | Rate | Lower bound | Upper bound |
| --- | --- | --- | --- |
| 0- 14 | 0 | 0 | 0 |
| 15- 19 | 0 | 0 | 0 |
| 20- 24 | 0 | 0 | 0.1 |
| 25- 29 | 0.1 | 0.1 | 0.2 |
| 30- 34 | 0.4 | 0.3 | 0.5 |
| 35- 39 | 0.9 | 0.8 | 1.0 |
| 40- 44 | 1.6 | 1.5 | 1.8 |
| 45- 49 | 2.5 | 2.4 | 2.7 |
| 50- 54 | 2.9 | 2.8 | 3.1 |
| 55- 59 | 3.3 | 3.1 | 3.4 |
| 60- 64 | 3.9 | 3.7 | 4.1 |
| 65- 69 | 4.7 | 4.4 | 4.9 |
| 70- 74 | 6.4 | 6.1 | 6.8 |
| 75- 79 | 8.4 | 8.0 | 8.8 |
| 80- 84 | 10.6 | 10.1 | 11.1 |
| 85+ | 12.8 | 12.3 | 13.3 |

**Table J.** Annual vaginal cancer incidence rates (per 100,000) [60,61]

| Age (years) | Rate | Lower bound | Upper bound |
| --- | --- | --- | --- |
| 0- 14 | 0 | 0 | 0 |
| 15- 19 | 0 | 0 | 0 |
| 20- 24 | 0 | 0 | 0 |
| 25- 29 | 0 | 0 | 0 |
| 30- 34 | 0.1 | 0 | 0.1 |
| 35- 39 | 0.1 | 0.1 | 0.2 |
| 40- 44 | 0.3 | 0.2 | 0.3 |
| 45- 49 | 0.4 | 0.4 | 0.5 |
| 50- 54 | 0.6 | 0.6 | 0.7 |
| 55- 59 | 0.8 | 0.7 | 0.9 |
| 60- 64 | 1.0 | 0.9 | 1.2 |
| 65- 69 | 1.3 | 1.2 | 1.4 |
| 70- 74 | 1.7 | 1.6 | 1.9 |
| 75- 79 | 2.1 | 1.9 | 2.3 |
| 80- 84 | 2.5 | 2.3 | 2.8 |
| 85+ | 2.9 | 2.6 | 3.2 |

**Table K.** Annual penile cancer incidence rates (per 100,000) [60,61]

| Age (years) | Rate | Lower bound | Upper bound |
| --- | --- | --- | --- |
| 0- 14 | 0 | 0 | 0 |
| 15- 19 | 0 | 0 | 0 |
| 20- 24 | 0 | 0 | 0 |
| 25- 29 | 0 | 0 | 0.1 |
| 30- 34 | 0.1 | 0.1 | 0.2 |
| 35- 39 | 0.2 | 0.2 | 0.2 |
| 40- 44 | 0.4 | 0.3 | 0.5 |
| 45- 49 | 0.5 | 0.5 | 0.6 |
| 50- 54 | 0.8 | 0.7 | 0.9 |
| 55- 59 | 1.2 | 1.1 | 1.3 |
| 60- 64 | 1.8 | 1.6 | 1.9 |
| 65- 69 | 2.6 | 2.4 | 2.8 |
| 70- 74 | 3.5 | 3.3 | 3.8 |
| 75- 79 | 4.4 | 4.1 | 4.8 |
| 80- 84 | 5.1 | 4.7 | 5.5 |
| 85+ | 6.3 | 5.8 | 6.9 |

**Table L.** Annual anal cancer incidence rates, males (per 100,000) [60,61]

| Age (years) | Rate | Lower bound | Upper bound |
| --- | --- | --- | --- |
| 0- 14 | 0 | 0 | 0 |
| 15- 19 | 0 | 0 | 0 |
| 20- 24 | 0 | 0 | 0 |
| 25- 29 | 0.1 | 0.1 | 0.1 |
| 30- 34 | 0.2 | 0.2 | 0.2 |
| 35- 39 | 0.6 | 0.5 | 0.7 |
| 40- 44 | 1.5 | 1.4 | 1.6 |
| 45- 49 | 2.1 | 2.0 | 2.2 |
| 50- 54 | 2.4 | 2.3 | 2.5 |
| 55- 59 | 2.5 | 2.4 | 2.7 |
| 60- 64 | 2.6 | 2.4 | 2.8 |
| 65- 69 | 2.9 | 2.7 | 3.2 |
| 70- 74 | 2.8 | 2.5 | 3.0 |
| 75- 79 | 2.8 | 2.5 | 3.0 |
| 80- 84 | 2.9 | 2.6 | 3.3 |
| 85+ | 2.6 | 2.3 | 3.0 |

**Table M.** Annual anal cancer incidence rates, females (per 100,000) [60,61]

| Age (years) | Rate | Lower bound | Upper bound |
| --- | --- | --- | --- |
| 0- 14 | 0 | 0 | 0 |
| 15- 19 | 0 | 0 | 0 |
| 20- 24 | 0 | 0 | 0 |
| 25- 29 | 0 | 0 | 0 |
| 30- 34 | 0.2 | 0.1 | 0.2 |
| 35- 39 | 0.5 | 0.4 | 0.6 |
| 40- 44 | 1.3 | 1.2 | 1.4 |
| 45- 49 | 2.8 | 2.6 | 2.9 |
| 50- 54 | 4.3 | 4.1 | 4.5 |
| 55- 59 | 4.9 | 4.7 | 5.1 |
| 60- 64 | 4.9 | 4.7 | 5.1 |
| 65- 69 | 4.9 | 4.7 | 5.2 |
| 70- 74 | 5.3 | 5.0 | 5.6 |
| 75- 79 | 5.1 | 4.8 | 5.4 |
| 80- 84 | 5.1 | 4.8 | 5.5 |
| 85+ | 4.5 | 4.2 | 4.8 |

**Table N.** Annual oropharyngeal cancer incidence rates, males (per 100,000) [60,61]

| Age (years) | Rate | Lower bound | Upper bound |
| --- | --- | --- | --- |
| 0- 14 | 0 | 0 | 0 |
| 15- 19 | 0 | 0 | 0 |
| 20- 24 | 0 | 0 | 0.1 |
| 25- 29 | 0.1 | 0.1 | 0.1 |
| 30- 34 | 0.3 | 0.2 | 0.3 |
| 35- 39 | 1.2 | 1.1 | 1.3 |
| 40- 44 | 3.9 | 3.8 | 4.1 |
| 45- 49 | 9.8 | 9.5 | 10.0 |
| 50- 54 | 17.4 | 17.0 | 17.7 |
| 55- 59 | 23.6 | 23.1 | 24.0 |
| 60- 64 | 24.8 | 24.3 | 25.3 |
| 65- 69 | 23.4 | 22.8 | 23.9 |
| 70- 74 | 20.3 | 19.7 | 21.0 |
| 75- 79 | 15.9 | 15.3 | 16.6 |
| 80- 84 | 13.0 | 12.4 | 13.7 |
| 85+ | 8.5 | 7.9 | 9.2 |

**Table O.** Annual oropharyngeal cancer incidence rates, females (per 100,000) [60,61]

| Age (years) | Rate | Lower bound | Upper bound |
| --- | --- | --- | --- |
| 0- 14 | 0 | 0 | 0 |
| 15- 19 | 0 | 0 | 0 |
| 20- 24 | 0 | 0 | 0.1 |
| 25- 29 | 0.1 | 0 | 0.1 |
| 30- 34 | 0.2 | 0.1 | 0.2 |
| 35- 39 | 0.4 | 0.4 | 0.5 |
| 40- 44 | 0.9 | 0.8 | 1.0 |
| 45- 49 | 1.9 | 1.8 | 2.0 |
| 50- 54 | 3.2 | 3.0 | 3.3 |
| 55- 59 | 4.1 | 3.9 | 4.3 |
| 60- 64 | 4.8 | 4.6 | 5.0 |
| 65- 69 | 5.3 | 5.1 | 5.6 |
| 70- 74 | 5.3 | 5.0 | 5.6 |
| 75- 79 | 5.0 | 4.7 | 5.3 |
| 80- 84 | 4.2 | 3.9 | 4.5 |
| 85+ | 3.2 | 3.0 | 3.5 |

**Table P.** Annual genital warts incidence rates, males (per person) [60,61]

| Age (years) | Rate | Lower bound | Upper bound |
| --- | --- | --- | --- |
| 0- 14 | 0.00011 | 0.00003 | 0.00041 |
| 15- 19 | 0.00074 | 0.00051 | 0.00065 |
| 20- 24 | 0.00236 | 0.00176 | 0.00293 |
| 25- 29 | 0.00272 | 0.00207 | 0.00501 |
| 30- 34 | 0.00223 | 0.00183 | 0.00388 |
| 35- 39 | 0.00223 | 0.00183 | 0.00252 |
| 40- 44 | 0.00118 | 0.00094 | 0.00189 |
| 45- 49 | 0.00118 | 0.00094 | 0.00128 |
| 50- 54 | 0.00092 | 0.00071 | 0.00118 |
| 55- 59 | 0.00092 | 0.00071 | 0.00086 |
| 60- 64 | 0.00048 | 0.00028 | 0.00100 |
| 65- 69 | 0.00048 | 0.00024 | 0.00087 |
| 70- 74 | 0.00043 | 0.00020 | 0.00087 |
| 75- 79 | 0.00043 | 0.00020 | 0.00087 |
| 80- 84 | 0.00024 | 0.00008 | 0.00087 |
| 85+ | 0.00024 | 0.00008 | 0.00087 |

**Table Q.** Annual genital warts incidence rates, females (per person) [60,61]

| **Age (years)** | **Rate** | **Lower bound** | **Upper bound** |
| --- | --- | --- | --- |
| 0- 14 | 0.00013 | 0.00004 | 0.00043 |
| 15- 19 | 0.00223 | 0.00176 | 0.00287 |
| 20- 24 | 0.00459 | 0.00356 | 0.00620 |
| 25- 29 | 0.00272 | 0.00195 | 0.00394 |
| 30- 34 | 0.00150 | 0.00119 | 0.00265 |
| 35- 39 | 0.00150 | 0.00119 | 0.00199 |
| 40- 44 | 0.00108 | 0.00081 | 0.00139 |
| 45- 49 | 0.00108 | 0.00081 | 0.00144 |
| 50- 54 | 0.00073 | 0.00052 | 0.00092 |
| 55- 59 | 0.00073 | 0.00052 | 0.00086 |
| 60- 64 | 0.00062 | 0.00035 | 0.00076 |
| 65- 69 | 0.00062 | 0.00029 | 0.00055 |
| 70- 74 | 0.00045 | 0.00018 | 0.00055 |
| 75- 79 | 0.00045 | 0.00018 | 0.00055 |
| 80- 84 | 0.00016 | 0.00001 | 0.00055 |
| 85+ | 0.00016 | 0.00001 | 0.00055 |

**Table R.** Percent of cervical, vulvar, vaginal, penile, anal and oropharyngeal cancers attributable to HPV types [60,61]

| **HPV Type** | **Cervical** | **Vulvar** | **Vaginal** | **Penile** | **Anal** | | **Oropharyngeal** | |
| --- | --- | --- | --- | --- | --- | --- | --- | --- |
|  |  |  |  |  | Male | Female | Male | Female |
| 16 | 50.1  (46.6 - 53.6) | 48.1  (40.8 - 55.4) | 53.4  (40.9 - 59.5) | 45.2  (34.7 - 56.1) | 75.3  (62.3 - 79.3) | 78.5  (69.1 - 80.9) | 61.6  (56.9 - 66.0) | 48.4  (40.6 - 56.4) |
| 18 | 16.1  (13.7 - 18.8) | 0.6  (0.1 - 3.2) | 1.7  (0.3 - 5.4) | 2.7  (0.8 - 9.0) | 3.8  (1.1 - 7.5) | 1.1  (0.2 - 2.7) | 1.8  (0.9 - 3.6) | 2.4  (0.9 - 6.2) |
| 31 | 2.1  (1.3 - 3.3) | 1.1  (0.3 - 4.0) | 0 | 0 | 0 | 1.2  (0.2 - 2.8) | 0.0  (0.0 - 0.9) | 0.7  (0.1 - 3.7) |
| 33 | 3.5  (2.4 - 5.0) | 9.3  (5.8 - 14.5) | 11.6  (5.7 - 17.0) | 5.1  (2.0 - 12.3) | 1.9  (0.3 - 5.2) | 8.4  (4.3 - 10.9) | 2.8  (1.6 - 4.8) | 8.8  (5.3 - 14.5) |
| 45 | 5.5  (4.1 - 7.3) | 0.6  (0.1 - 3.1) | 3.3  (0.9 - 7.4) | 2.7  (0.8 - 9.0) | 0 | 0 | 0.7  (0.2 - 2.0) | 0 |
| 52 | 1.8  (1.1 - 3.1) | 2.7  (1.1 - 6.2) | 1.7  (0.3 - 5.4) | 1.3  (0.2 - 6.8) | 1.9  (0.3 - 5.2) | 0 | 0.7  (0.2 - 2.0) | 0 |
| 58 | 1.8  (1.1 - 3.0) | 0.6  (0.1 - 3.1) | 1.7  (0.3 - 5.4) | 0 | 0  (0.0 - 2.8) | 1.1  (0.2 - 2.7) | 0.2  (0.0 - 1.3) | 0 |

**Table S.** Base case estimates and ranges of the cost per case of non-cervical HPV-related diseases (2018 U.S. dollars) [60,61]

|  | **Median cost estimate** | | |
| --- | --- | --- | --- |
|  | **Base case value** | **Lower bound** | **Upper bound** |
| Anal cancer | $52,600 | $40,400 | $78,300 |
| Vaginal cancer | $111,400 | $30,200 | $142,400 |
| Vulvar cancer | $41,300 | $26,300 | $58,700 |
| Oropharyngeal cancer | $141,800 | $82,000 | $166,600 |
| Penile cancer | $22,100 | $10,900 | $43,300 |
| Genital warts | $680 | $340 | $770 |

**Table T.** Quality of life detriments for treatment for non-cervical HPV-associated diseases [60,61]

|  | **Base case** | **Lower bound** | **Upper bound** |
| --- | --- | --- | --- |
| Vaginal cancer | 0.32 | 0.16 | 0.52 |
| Vulvar cancer | 0.32 | 0.16 | 0.52 |
| Penile cancer | 0.29 | 0.20 | 0.38 |
| Anal cancer | 0.51 | 0.21 | 0.83 |
| Oropharyngeal cancer | 0.25 | 0.20 | 0.30 |
| Genital warts | 0.024 | 0.008 | 0.100 |

**Table U.** Relative 5-year cancer survival probabilities [31,60,61]

|  | **Age < 50 years** | | | **Age 50 years and over** | | |
| --- | --- | --- | --- | --- | --- | --- |
|  | **Base case** | **Lower bound** | **Upper bound** | **Base case** | **Lower bound** | **Upper bound** |
| Vaginal | 0.705 | 0.592 | 0.792 | 0.524 | 0.470 | 0.575 |
| Vulvar | 0.844 | 0.805 | 0.875 | 0.632 | 0.605 | 0.658 |
| Penile | 0.755 | 0.662 | 0.826 | 0.667 | 0.621 | 0.708 |
| Anal, female | 0.774 | 0.732 | 0.810 | 0.723 | 0.698 | 0.747 |
| Anal, male | 0.629 | 0.584 | 0.670 | 0.654 | 0.618 | 0.688 |
| Oropharyngeal, female | 0.634 | 0.580 | 0.683 | 0.560 | 0.533 | 0.586 |
| Oropharyngeal, male | 0.743 | 0.721 | 0.764 | 0.635 | 0.622 | 0.647 |

**Table V. Harvard vaccine uptake rates in females (current “status quo” scenario)*** [66-71]

| **Age** | **2007** | **2008+** |
| --- | --- | --- |
| <12 | 0.000 | 0.000 |
| 12 | 0.295 | 0.295 |
| 13 | 0.145 | 0.129 |
| 14 | 0.166 | 0.129 |
| 15 | 0.185 | 0.129 |
| 16 | 0.188 | 0.129 |
| 17 | 0.209 | 0.129 |
| 18 | 0.209 | 0.129 |
| 19-26 | 0.026 | 0.026 |
| 27+ | 0.000 | 0.000 |

* Values represent proportion newly vaccinated in each year (i.e., among those not previously vaccinated).

**Table W. Harvard vaccine uptake rates in males (current “status quo” scenario)*** [68-71]

| **Age** | **2010** | **2011+** |
| --- | --- | --- |
| <12 | 0.000 | 0.000 |
| 12 | 0.249 | 0.249 |
| 13 | 0.145 | 0.097 |
| 14 | 0.166 | 0.097 |
| 15 | 0.185 | 0.097 |
| 16 | 0.188 | 0.097 |
| 17 | 0.209 | 0.097 |
| 18 | 0.209 | 0.097 |
| 19-26 | 0.026 | 0.019 |
| 27+ | 0.000 | 0.000 |

* Values represent proportion newly vaccinated in each year (i.e., among those not previously vaccinated).

**Table X. Policy1-Cervix vaccine uptake rates in females (current “status quo” scenario)*** [66-71]

| **Age** | **2007** | **2008** | **2009** | **2010** | **2011** | **2012** | **2013** | **2014** | **2015** | **2016+** |
| --- | --- | --- | --- | --- | --- | --- | --- | --- | --- | --- |
| <12 | 0.000 | 0.000 | 0.000 | 0.000 | 0.000 | 0.000 | 0.000 | 0.000 | 0.000 | 0.000 |
| 12 | 0.000 | 0.000 | 0.000 | 0.000 | 0.000 | 0.000 | 0.000 | 0.000 | 0.000 | 0.295 |
| 13 | 0.145 | 0.195 | 0.232 | 0.229 | 0.202 | 0.258 | 0.262 | 0.295 | 0.288 | 0.129 |
| 14 | 0.166 | 0.102 | 0.137 | 0.078 | 0.075 | 0.149 | 0.136 | 0.150 | 0.126 | 0.129 |
| 15 | 0.185 | 0.109 | 0.113 | 0.105 | 0.086 | 0.150 | 0.134 | 0.128 | 0.102 | 0.129 |
| 16 | 0.188 | 0.180 | 0.151 | 0.119 | 0.021 | 0.121 | 0.073 | 0.051 | 0.106 | 0.129 |
| 17 | 0.209 | 0.158 | 0.064 | 0.120 | 0.075 | 0.149 | 0.139 | 0.189 | 0.179 | 0.129 |
| 18 | 0.209 | 0.129 | 0.129 | 0.129 | 0.129 | 0.129 | 0.129 | 0.129 | 0.129 | 0.129 |
| 19-26 | 0.026 | 0.026 | 0.026 | 0.026 | 0.026 | 0.026 | 0.026 | 0.026 | 0.026 | 0.026 |
| 27+ | 0.000 | 0.000 | 0.000 | 0.000 | 0.000 | 0.000 | 0.000 | 0.000 | 0.000 | 0.000 |

* Values represent proportion newly vaccinated in each year (i.e., among those not previously vaccinated).

**Table Y. Policy1-Cervix vaccine uptake rates in males (current “status quo” scenario)*** [68-71]

| **Age** | **2010** | **2011** | **2012** | **2013** | **2014** | **2015** | **2016+** |
| --- | --- | --- | --- | --- | --- | --- | --- |
| <12 | 0.000 | 0.000 | 0.000 | 0.000 | 0.000 | 0.000 | 0.000 |
| 12 | 0.000 | 0.000 | 0.000 | 0.000 | 0.000 | 0.000 | 0.249 |
| 13 | 0.016 | 0.066 | 0.117 | 0.162 | 0.249 | 0.252 | 0.097 |
| 14 | 0.018 | 0.044 | 0.075 | 0.104 | 0.137 | 0.092 | 0.097 |
| 15 | 0.009 | 0.064 | 0.100 | 0.131 | 0.097 | 0.050 | 0.097 |
| 16 | 0.013 | 0.051 | 0.061 | 0.090 | 0.076 | 0.106 | 0.097 |
| 17 | 0.010 | 0.061 | 0.097 | 0.111 | 0.077 | 0.032 | 0.097 |
| 18 | 0.010 | 0.097 | 0.097 | 0.097 | 0.097 | 0.097 | 0.097 |
| 19-21 | 0.019 | 0.019 | 0.019 | 0.019 | 0.019 | 0.019 | 0.019 |
| 22+ | 0.000 | 0.000 | 0.000 | 0.000 | 0.000 | 0.000 | 0.000 |

* Values represent proportion newly vaccinated in each year (i.e., among those not previously vaccinated).

**Table Z. Utilities for cervical screening and cancer**

| **Heath state or event** | **Harvard**  **(duration)** | **Policy1-Cervix**  **(duration)** |
| --- | --- | --- |
| Abnormal test result and/or colposcopy visit | No decrement | 0.994 [76]  (1 year) |
| Treatment for precancerous lesion | No decrement | 0.99 [76]  (1 year) |
| Cancer (local) | 0.68 [77,78]  (10 years, or until death) | 0.68 [77,78]  (10 years, or until death) |
| Cancer (regional) | 0.56 [77,78]  (10 years, or until death) | 0.56 [77,78]  (10 years, or until death) |
| Cancer (distant) | 0.48 [78]  (10 years, or until death) | 0.48 [78]  (10 years, or until death) |

**Table AA.** Proportion (%) of population screening at different frequencies [57]

| **Frequency** | **% Women** |
| --- | --- |
| 1-year | 9.3 |
| 2-year | 16.2 |
| 3-year | 10.6 |
| 4-year | 35.2 |
| 5-year | 14.4 |
| none | 14.4 |

**Table BB.** Proportion of women who received colposcopy/biopsy, by cytology result [59]

| **Cytology Result** | **% Received colposcopy/biopsy** |  |
| --- | --- | --- |
| Negative | 0.013 |  |
| ASC-US | 0.494 |  |
| ASC-H | 0.623 |  |
| LSIL | 0.507 |  |
| HSIL | 0.760 |  |
| Abbreviations: ASC-US, atypical squamous cells of undetermined significance; ASC-H, atypical squamous cells indicating high-grade; HSIL, high-grade squamous intraepithelial lesions; LSIL, low-grade squamous intraepithelial lesions. | | |

**Table CC.** Proportion of women who received precancer treatment, by histology result [59]

| **Histology Result** | **Compliance** |
| --- | --- |
| Negative | 0.016 |
| CIN2 | 0.473 |
| CIN3 | 0.630 |
| Cancer | 0.132 |

Abbreviations: CIN2, cervical intraepithelial neoplasia, grade 2; CIN3, cervical intraepithelial neoplasia, grade 3.

**Table DD. Incremental costs and QALYs associated with HPV vaccination strategies (base-case cost-effectiveness analysis)***

| **Strategies** | **Harvard** | | **Policy1-Cervix** | |
| --- | --- | --- | --- | --- |
| Cervical outcomes only † | Incremental  cost | Incremental QALY | Incremental  cost | Incremental QALY |
| Vacc to age 30 ‡ | $4,561,890,500 | 2,770 | $4,468,048,100 | 6,380 |
| Vacc to age 35 | $3,832,955,900 | 3,650 | $3,752,384,800 | 7,490 |
| Vacc to age 40 | $4,222,345,300 | 4,970 | $4,107,960,900 | 7,670 |
| Vacc to age 45 | $4,571,401,800 | 5,610 | $4,343,228,300 | 12,840 |
| All outcomes § | | | | |
| Vacc to age 30 ‡ | $4,481,889,900 | 7,140 | $4,379,441,000 | 12,840 |
| Vacc to age 35 | $3,745,211,700 | 8,800 | $3,697,872,100 | 11,330 |
| Vacc to age 40 | $4,120,855,000 | 11,040 | $4,052,371,100 | 11,430 |
| Vacc to age 45 | $4,480,097,500 | 11,210 | $4,287,646,000 | 16,400 |

* Values represent incremental costs and incremental effects, in terms of quality-adjusted life years (QALYs), compared to the next less costly strategy; values are rounded to nearest hundreds (costs) or tens (QALYs). Estimates reflect differences in lifetime health and economic consequences aggregated across multiple birth cohorts in the U.S. population over years 2019-2119. The underlying population structure by age from years 2019 onward was obtained from the United Nations World Population Prospects: The 2017 Revision (United Nations, Department of Economic and Social Affairs, Population Division (2017). World Population Prospects: The 2017 Revision, DVD Edition).

† Only outcomes related to cervical cancer were included.

‡ Vaccination to age 30 was compared against current vaccination.

§ All HPV-related outcomes (cervical, anal, oropharyngeal, vulvar, vaginal, and penile cancers, as well as genital warts) were included.

**Figure A.** Schematic of Harvard HPV transmission model


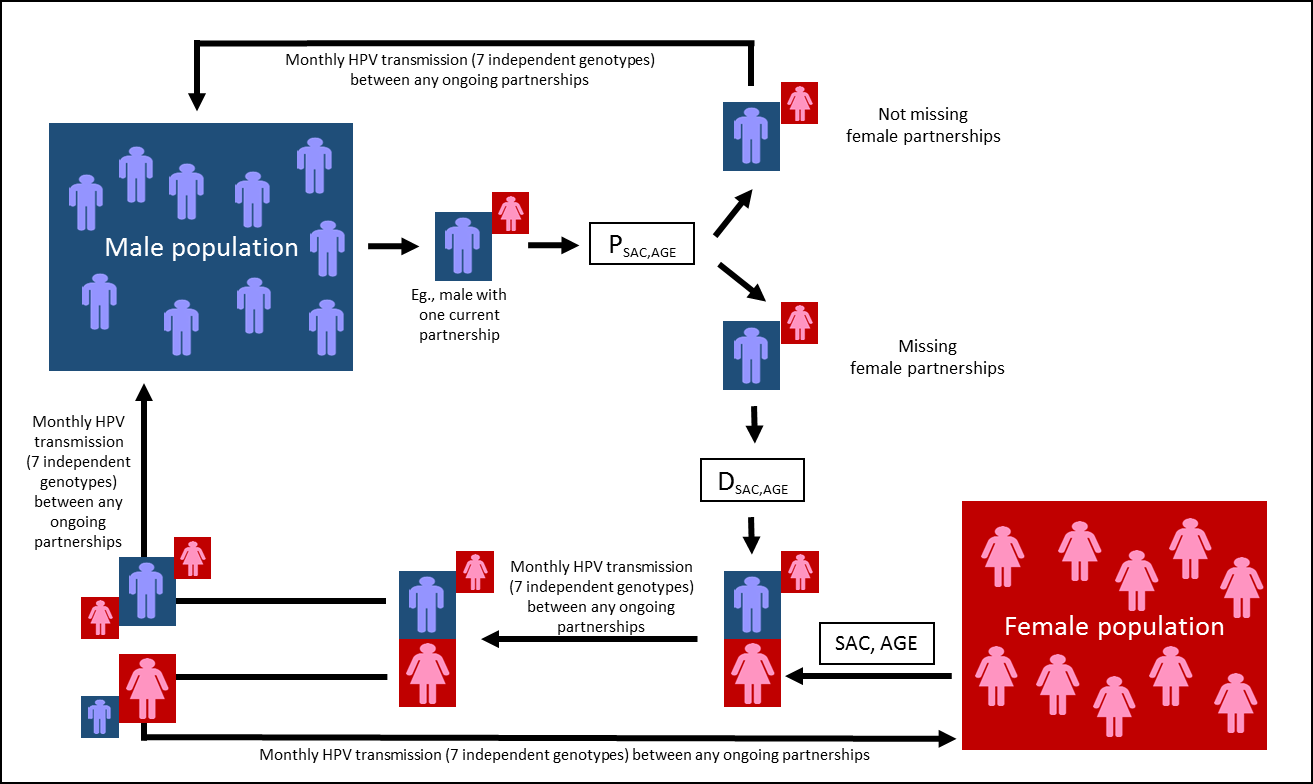


**Figure B.** Schematic of Harvard cervical cancer natural history model

**
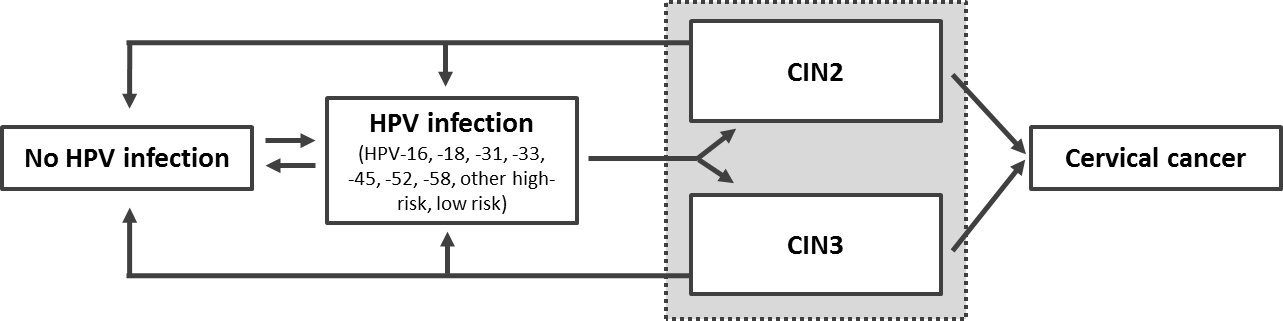
**

**Figure C.**  Schematic of Policy1-Cervix


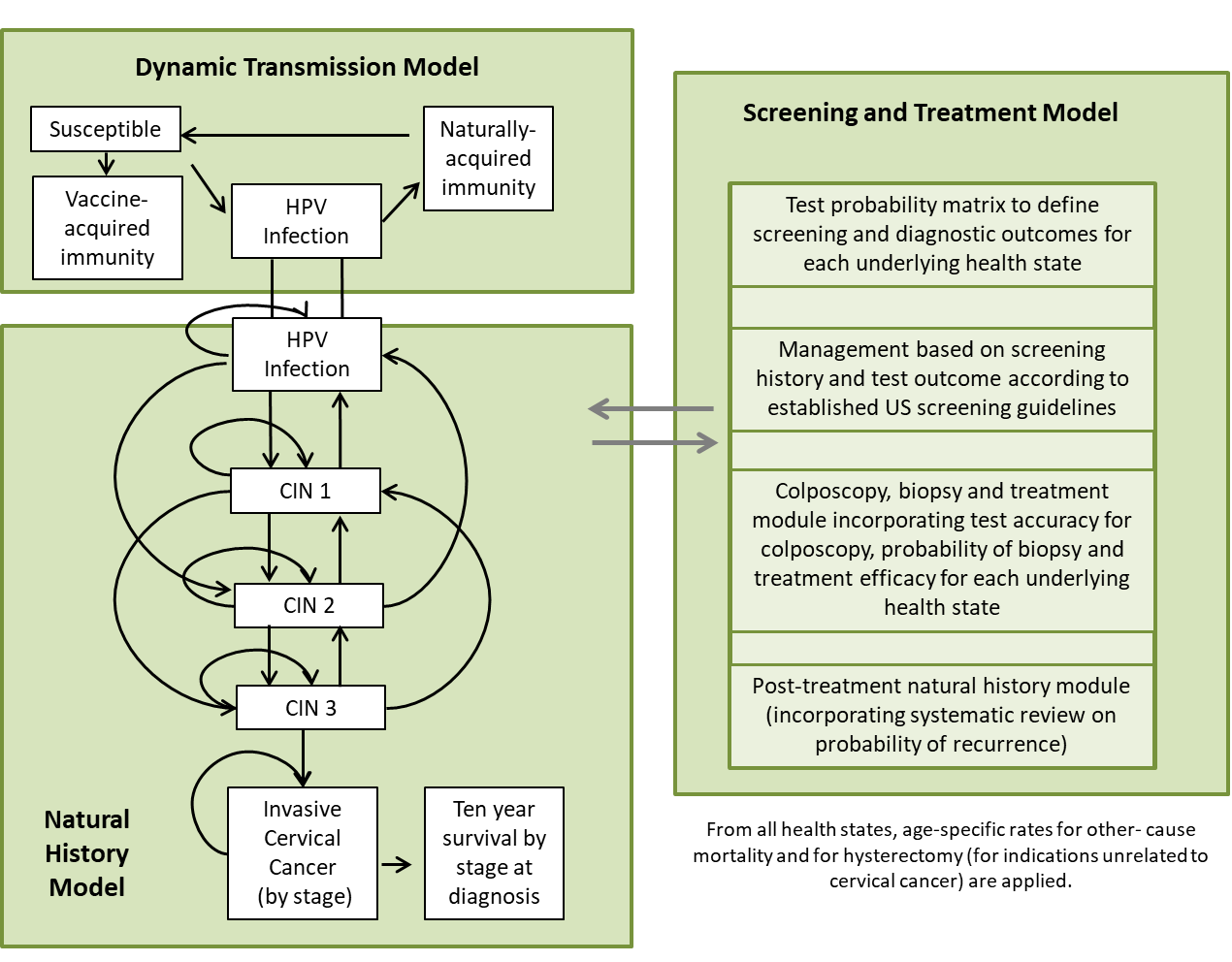


**Figure D.** Calibration results: Model fit to HPV prevalence

Harvard

Policy1-Cervix

Data (95% CI) [21]

**HPV 16**

**HPV 18**

**HPV Prevalence**

**Other HPV**

**HPV 31/33/45/52/58**

**HPV Prevalence**

**Age**

**Age**

**Figure E.** Calibration results: Model fit to HPV type distribution

Harvard

Policy1-Cervix

Data (95% CI) [55]

**HPV 16**

**HPV 18**

**HPV 31/33/45/52/58**

**% HPV**

**Age**

**Age**

**% HPV**

**Other HR**

**Figure F.** Validation results: Model fit to cervical cancer incidence rates, pre-screening

**Cervical cancer incidence** (per 100,000 women)

Harvard

Policy1-Cervix

CTR, 1950-1959 [56]

CTR, 1960-1969 [56]

**Age**

**Figure G.** Validation results: Model fit to cervical cancer mortality rates, pre-screening

**Cervical cancer mortality** (per 100,000 women)

Harvard

Policy1-Cervix

CTR, 1950-1959 [56]

CTR, 1960-1969 [56]

**Age**

Figure H. Validation results: Cervical cancer incidence rates by age under assumptions of imperfect screening and follow-up compliance, compared to SEER data [31]

**Cervical cancer incidence** (per 100,000 women)

**Age**

**Figure I.** Validation results: Cervical cancer mortality rates by age under assumptions of imperfect screening and follow-up compliance, compared to SEER data [31]

**Age**

**Cervical cancer mortality** (per 100,000 women)
